# Enhanced drought tolerance and photosynthetic efficiency in *Arabidopsis* by overexpressing phosphoenolpyruvate carboxylase from a single-cell C4 halophyte *Suaeda aralocaspica*

**DOI:** 10.3389/fpls.2024.1443691

**Published:** 2024-08-30

**Authors:** Caixia Li, Juan Wang, Haiyan Lan, Qinghui Yu

**Affiliations:** ^1^ Xinjiang Key Laboratory of Biological Resources and Genetic Engineering, College of Life Science and Technology, Xinjiang University, Urumqi, China; ^2^ Institute of Horticulture Crops, Xinjiang Academy of Agricultural Sciences, Urumqi, China

**Keywords:** drought, PEPC, photosynthesis, single-cell C4 cycle, *Suaeda aralocaspica*

## Abstract

In crop genetic improvement, the introduction of C4 plants’ characteristics, known for high photosynthetic efficiency and water utilization, into C3 plants has been a significant challenge. This study investigates the effects of the desert halophyte *Suaeda aralocaspica SaPEPC1* gene from a single-cell C4 photosythetic pathway, on drought resistance and photosynthetic performance in *Arabidopsis*. We used transgenic *Arabidopsis* with *Zea mays ZmPEPC1* from C4 plant with classic Kranz anatomical structure and *Arabidopsis AtPEPC1* from C3 photosynthetic cycle plants as controls. The results demonstrated that C4 photosynthetic-type *PEPCs* could improve drought resistance in plants through stomatal closure, promoting antioxidant enzyme accumulation, and reducing reactive oxygen species (ROS) accumulation. Overexpression of *SaPEPC1* was significantly more effective than *ZmPEPC1* in enhancing drought tolerance. Notably, overexpressed *SaPEPC1* significantly improved light saturation intensity, electron transport rate (ETR), photosynthetic rate (Pn), and photoprotection ability under intense light. Furthermore, overexpression *SaPEPC1* or *ZmPEPC1* enhanced the activity of key C4 photosynthetic enzymes, including phosphoenolpyruvate carboxylase (PEPC), pyruvate orthophosphate dikinase (PPDK) and NADP-malic enzyme (NADP-ME), and promoted photosynthetic product sugar accumulation. However, with *AtPEPC1* overexpression showing no obvious improvement effect on drought and photosynthetic performance. Therefore, these results indicated that introducing C4-type *PEPC* into C3 plants can significantly enhance drought resistance and photosynthetic performance. However, *SaPEPC1* from a single-cell C4 cycle plant exhibits more significant effect in ETR and PSII photosynthesis performance than *ZmPEPC1* from a classical C4 anatomical structure plant, although the underlying mechanism requires further exploration.

## 1 Introduction

The photosynthesis with which a plant captures light energy and converts it to chemical energy remains a key determinant in the nature (Simkin et al., 2019). The high yield of C4 (compared to C3) plants has spurred interest in exploring the mechanism of C4 photosynthetic pathways (Caemmerer and Furbank, 2016), in which hundreds of genes are involved (Berry et al., 2013). The potential for yield in the C3 plant rice is limited by the photosynthetic capacity of the leaves, and the goal of the international C4 Rice Consortium is to identify and engineer the genes required for C4 photosynthesis in rice to ensure the C4 photosynthetic pathway works in rice, however, the biggest challenge is to generate two-celled C4 shuttles in rice (Caemmerer et al., 2012; Kajala et al., 2011). In addition, understanding the the kinetic properties of the introduced enzymes, PEP transport activity and metabolite interactions is also a major challenge in the development of C4 rice (Miyao et al., 2011). It was found that the chenopodiaceae terrestrial plants *Bienertia cycloptera*, *B. sinuspersici*, *B. kavirense* and *Suaeda aralocaspica* could complete the C4 photosynthetic cycle in one cell through spatial partitioning of organelles and key enzymes in distinct cytoplasmic domains within single chlorenchyma cells. And the photosynthetic efficiency is equals to that of C4 with Kranz structure (Caemmerer et al., 2014; Lung et al., 2012). Moreover, it was also found in aquatic plant *Hydrilla verticillata* that C4 photosynthesis cycle can also be completed in a single cell, so it is of great significance to excavate the key genes for improving photosynthesis from plants that complete C4 photosynthesis in a single cell (Mukundan et al., 2024; Bowes et al., 2002). This provides a new way to introduce C4 photosynthetic traits into C3 plants.

C4 photosynthesis is a complex process. With the development of omics and molecular biology, several important photosynthetic genes have been identified (Cui, 2021). In *Alloteropsis semialata*, the intermediates with a weak C4 photosynthetic cycle were distinguished from the C3 phenotype by upregulating 73 genes, including those encoding four core C4 enzymes: aspartate aminotransferase (AST, EC2.6.1.1), phosphoenolpyruvate carboxykinase (PEPCK, EC4.1.1.49), phosphoenolpyruvate carboxylase (PEPC), and pyruvate orthophosphate dikinase (PPDK) (Dunning et al., 2019). This finding suggests that the key C4 photosynthetic enzymes are essential for high performance of the photosynthesis. Improving carbon concentration to mitigate plant respiration holds particular significance in this regard (Faralli and Lawson, 2020). Notably, PEPC has garnered much attention for its high CO_2_ fixation efficiency in C4 and CAM photosynthetic cycles in this purpose (Endo et al., 2008).The cytosolic enzyme PEPC is widely distributed in higher plants, algae, and microorganisms (Doubnerová and Ryšlavá, 2011). Current research emphasizes incorporating genes from the classical C4 photosynthetic cycle (e.g., maize, sugarcane) into C3 plants to enhance photosynthetic performance (Giuliani et al., 2019). Studies found that introducing maize *PEPC*, *PPDK*, or both into C3 crop wheat results in improved photosynthetic performance and yield characteristics. Interestingly, the exogenous C4- *PEPC* gene is known to be more effective than *PPDK*, with the combination of both genes showing synergistic effects (Zhang et al., 2014). Additionally, overexpression of ZmPEPC in wheat increased the content of proline and soluble sugar in wheat, thus improving the drought resistance of transgenic wheat (Qin et al., 2016). So far, most studies have focused on the role of classical C4 plant *PEPC* genes in completing the C4 photosynthetic cycle, with limited attention being given to the single-cell C4 photosynthetic cycle associated *PEPC*s.

*S. aralocaspica* is a unique C4 plant by completing the C4 photosynthetic cycle in a single, slender chlorenchyma cell, in which ribulose 1,5-bisphosphate carboxylase/oxygenase (Rubisco) containing chloroplasts and NAD-malic enzyme (NAD-ME) containing mitochondria are concentrated proximally, and PPDK containing chloroplasts and cytosolic PEPC evenly dispersed distally (Voznesenskaya et al., 2001). The organellar distribution of photosynthetic enzymes at opposite ends of the chlorenchyma cell is similar to the Kranz anatomy (Lung et al., 2012). Studies have demonstrated that PEPC from *S. aralocaspica* shares kinetic and regulatory characteristics with other C4 species, exhibiting significantly higher activity than C3 species, which may be necessary for completing the single-celled C4 photosynthetic cycle (Lara et al., 2006). So far, two plant types (*SaPEPC1* and *SaPEPC2*) and a bacterial type (*SaPEPC4*) of *PEPC* in *S. aralocaspica* have been identified, and the *SaPEPC1* is more active in chlorenchyma cell development and response to light variations, which suggests a determinant role of *SaPEPC1* in photosynthesis (Cheng et al., 2016; Cao et al., 2021). Based on these findings, a systematic study of the *SaPEPC1* gene function in improving photosynthetic performance and its distinctions from other photosynthetic *PEPC* types holds significance. For this purpose, this study evaluated the following aspects: (i) Effects of overexpressing *SaPEPC1* on drought resistance of *Arabidopsis*. (ii) Alterations in photosynthetic performance of transgenic *Arabidopsis* by overexpression of *SaPEPC1*. This study may enhance our understanding of *SaPEPC1*’s function in improving drought tolerance and photosynthesis efficiency, thereby offering a theoretical reference for genetic improvement of C3 crops’ yield.

## 2 Materials and methods

### 2.1 Plant growth and treatments

The wild type (WT) *Arabidopsis thaliana* used in this study was the ecotype Columbia-0 (Col-0). To obtain transgenic *Arabidopsis* we inserted the coding region of the gene (*SaPEPC*1, *ZmPEPC1*, *AtPEPC1*) into the plant expression vector pCAMBIA2300 driven by CaMV35S promoter using Kpn I and Sal I (or XbaI) cleavage sites, respectively, the recombinant plasmid was transformed into *Agrobacterium tumefaciens* strain EHA105, and then obtained the transgenic *Arabidopsis* by inflorescences. Transgenic *Arabidopsis* was identified by PCR, and the PCR reaction system was as follows: 94°C 5 min; 30 cycles of 94°C 30 s, 55°C 30 s, and 72°C 3 min; 72°C 10 min, the primer sequences were presented in **Supplementary Table S1**. Both WT and transgenic T3 generation seeds underwent surface sterilization (75% ethanol for 10 min, anhydrous ethanol for 2 min) and were planted in 1/2 Murashige and Skoog (MS) medium. Seeds were placed at 4°C for 2 d to break seed dormancy, then moved to a plant culture room at 22°C with a 16 h (light)/8 h (dark) cycle for approximately 10 d. The seedlings were transplanted into pots (6.5 × 6.5 × 8 cm, length × width × depth, each pot had four seedlings) containing a 3:1:1 mixture of peat soil: vermiculite: perlite (v/v), and grown under conditions of 22–25°C, 30 - 40% relative humidity, and a 16 h (light)/8 h (dark) photoperiod. For different light intensity treatment, using LED composite light (power 18 W, Zhong ke san an, China), three gradients were designed: normal light (200 μmol·m^-2^·s^-1^), intense light (440 μmol·m^-2^·s^-1^), and weak light (120 μmol·m^-2^·s^-1^). After about three weeks, functional leaves from the same position of the plants were frozen to measure the experimental indexes.

### 2.2 Seed germination

Intact and mature WT and T3 transgenic *Arabidopsis* seeds (close-maturity seeds) were selected for seed germination experiments. After surface sterilization, the seeds were evenly spread on 1/2 MS medium in Petri dishes with diameters of 9 cm, containing 0, 200, and 300 mM mannitol. The Petri dish was sealed with sealing film and incubated in a plant culture room at 22°C under a 16 h (light)/8 h (dark), with 30–40% relative humidity. Germination percentage was monitored every 24 h for 8 d. Each treatment had three replicates, with 30 seeds per replicate. The germination percentage was calculated using the formula: (m/n) × 100%, where *m* represents the number of germinated seeds and *n* is the total number of seeds.

### 2.3 Stomatal observation

Functional leaves aged 3–4 weeks were collected from the same leaf position the *Arabidopsis* plants. At 10 a.m., the lower epidermis was submerged downwards in stomatal opening buffer (50 mM KCl, 10 mM MES-KOH, pH 6.15, 100 μM CaCl_2_) for 2 h, followed by soaking in H_2_O and 300 mM mannitol for an additional 2 h. Subsequently, the lower epidermis was gently removed and mounted on a slide for observation under a light microscope (Leica D-35578, Wetzlar, Germany) and photographed using Leica Application Suite X. The stomatal width-to-length ratio was quantified using Image J software (National Institutes of Health, USA). Three leaves were checked per line of transgenic *Arabidopsis*, with a minimum of 30 clear stomata observed per leaf.

### 2.4 Measurement of the activity of antioxidant enzymes, the content of reactive oxygen species and lipid peroxidation

WT and transgenic *Arabidopsis* lines grown under LED white light (power 30 W, Jin xiang ya, China) for 3–4 weeks were selected and treated with 300 mM mannitol, using water as a control. After 24 h, leaves (0.1 g) from the same position of the plants were immediately placed in liquid nitrogen and stored at – 80°C. Antioxidant enzymes, ROS and MDA were quantified using a commercial kit (Comin, Suzhou, China), following the manufacturer’s protocol. The protein concentrations were determined using the Bradford protein assay kit (Coolaber, Beijing, China). Three biological replicates were employed for each assay.

The leaves (0.1 g) were homogenized with a mortar and pestle in ice-cold 0.2 M phosphate buffer (pH 7), which included 0.1 mM EDTA. The homogenate was centrifuged at 8000 g for 10 min at 4°C and the resulting supernatant was utilized for the determination of peroxides (POD, EC1.11.1.17, POD-1-Y), superoxide dismutase (SOD, EC 1.15.1.1, SOD-1-Y) and catalase (CAT, EC 1.11.1.6, CAT-1-Y). The solution for POD assay was prepared by combining ddH_2_O, 0.75% hydrogen peroxide (H_2_O_2_), guaiacol (20 mM), phosphate buffer (400 mM pH 7.5), and the enzyme extract. The addition of the enzyme extract initiated the reaction, and the absorption values were recorded at 470 nm after 1 and 2 minutes of reaction time (Ezzat et al., 2021).

The activity of SOD was determined by measuring the reduction of nitroblue tetrazolium (NBT). The reaction was conducted in sodium phosphate buffer (50 mM, pH 7.8) containing the enzyme extract, 2 μM riboflavin, 65 μM NBT, 13 μM methionine, and 1 μM ethylenediamine tetraacetic sodium. The solution was thoroughly mixed and incubated for 30 min sat room temperature, after which the absorbance was determined at 560 nm (Liu et al., 2014). For CAT, the reaction mixture comprised the enzyme extract, 50 mM phosphate buffer (pH 6.0) and 10 mM H_2_O_2_, and the initial and final (after 1 minute of reaction) absorbance were recorded at 240 nm (Liu et al., 2014).

To determine the activity of the ascorbate peroxidase (APX, EC1.1.11.1, APX-1-W), the homogenization of leaves (0.1 g) was done in 1 ml potassium phosphate buffer (50 mM), the homogenate was centrifuged at 13000 g for 20 min at 4°C, the supernatant was enzyme active extract. The APX assay buffer contained an enzyme extract, potassium phosphate buffer (200 mM), ascorbic acid (10 mM), and EDTA (0.5 M). Following rapid mixing, the absorbance of 10 s and 130 s was measured at 290 nm (Mughal et al., 2020).

To determine the content of the H_2_O_2_ (H_2_O_2_-1-Y), the leaves (0.1 g) were subjected to homogenization in cold acetone (1 ml). The homogenate was centrifuged at 8000 g for 10 min at 4°C, after which the supernatant was utilized for determination. The reaction mixture consisted of the supernatant, 20% titanium reagent and 17 M ammonia solution. The mixture was centrifuged at 4000 g at 25°C for 10 min and supernatant was discarded.the resulting precipitate was dissolved in a mixture of 0.1% titanium sulphate and 20% H_2_SO_4_ solution. Following a five-minute incubation period at room temperature, the absorbance at 415 nm was recorded (Esim et al., 2014).

To determine the concentration of superoxide anion (O_2_^-^, SA-1-G), leaves sample (0.1) was homogenized in 1 mL of 65 mM potassium phosphate buffer (pH 7.8), and subsequently centrifuged at 10000 g for 10 min in 4°C, to obtain the supernatant. The reaction amixture consisted of 65 mM potassium phosphate buffer (pH 7.8) and 10 mM hydroxylammonium chloride was incubated for 1 min at 25°C. Subsequently, 17 mM sulfanilic acid, 7 mM anaphthyl amine and the supernatant were added to the incubation mixture. Following 30 min reaction at 37°C the absorbance was measured at 530 nm (He et al., 2005).

Malondialdehyde (MDA, MDA-1-Y) is a product of membrane lipid peroxidation, and its concentration is positively correlated with the degree of membrane lipid peroxidation. Initially, 0.1 g of leaves were homogenized in 1 ml of 0.1 M trichloroacetic acid. The resulting mixture was subjected to centrifugation at 8000 g for 10 min at 4°C. The supernatant was colleted and mixed with 0.5% thiobarbituric acid and 20% trichloroacetic acid, which were ncubated at 95°C for 30 min, cooled in an ice bath, and then centrifuged at 10000 g for 10 min at 25°C. Absorbance readings were conducted at 532 and 600 nm (Amirikhah et al., 2019).

### 2.5 Chlorophyll fluorescence

The IMAGING-PAM chlorophyll fluorescence imaging system (Walz, Effeltrich, Germany) was utilized to examine chlorophyll fluorescence changes in functional leaves under varying light treatments. Initially, plants were dark-adapted for 30 min before measurement, with actinic light intensity set to 281 μmol·m^-2^·s^-1^ using the Imaging Win software (Walz, Effeltrich, Germany). Subsequently, the kinetics mode was employed to determine the relevant chlorophyll fluorescence parameters. Finally, the light curve mode was selected to establish a photosynthetically active radiation (PAR) gradient (from weak to strong) for measuring the photosynthetic electron transport rate. The rapid photoresponse curve was derived by fitting ETR data using the quadratic equation y=x/(a*x^2+b*x+c) in Origin Pro 9.1 software (Massachusetts, USA). Three plants (biological replicates) were sampled per transgenic line, with each plant checked at two sites within the functional leaf.

A Pocket PEA high-speed continuous excitation fluorometer (Hansatech, Norfolk, England) was used to measure the rapid chlorophyll fluorescence induction kinetics curve (OJIP curve). Leaves were dark-adapted using a clip and metal shutter closed for 30 min. The chlorophyll transient fluorescence was induced with red light (650 nm) at 3500 μmol·m^-2^·s^-1^ from three red diode spotlight sources, capturing complete OJIP curves within 1 s. The obtained OJIP curves were analyzed according to the method described by Strasser et al. (2010) on JIP test, and photosystem II (PSII) parameters derived from the OJIP transient were analyzed. **Supplementary Table S2** presents the chlorophyll fluorescence parameters. The relative variable fluorescence O to P was normalized using Vt = (Ft-Fo)/(Fm-Fo), where *Ft* represents fluorescence at any time, *Fo* is the minimum fluorescence, and *Fm* is the maximum fluorescence. The radar data processing formula utilized was C_test_/C_control_, with C_test_ as the experimental treatment group fluorescence parameter and C_control_ as the experimental control group’s fluorescence parameter. Six biological replicates were conducted per transgenic line.

### 2.6 Measurements of gas exchange parameters

The net photosynthetic rate (Pn), stomatal conductance (Gs), intercellular CO_2_ concentration (Ci) and transpiration rate were measured by CIRAS-3 portable photosynthesis system (PP-systems, USA). The measurement time was 9:00–13:00 O’clock, the light source was 1100 μmol·m^2^·s^-1^, the atmospheric CO_2_ concentration was 380 - 420 μmol mol^-1^, the leaf chamber temperature was 22-25°C, and the relative humidity was 40%. The largest, healthy leaf of a plant was selected for data collection by avoiding the main vein. Three out of 6 biological replicates for each transgenic line were used for plotting.

### 2.7 Measurement of enzymatic activities of photosynthesis and sugar synthesis

The activities of carbonic anhydrase (CA, EC4.2.1.1, BC5445), Rubisco (EC 4.1.1.39, BC0445), PEPC (EC 4.1.1.31, BC2195), NADP-ME (EC 1.1.1.40, BC1125), NADP-malate dehydrogenase (NADP-MDH, EC 1.1.1.37, BC1055), sucrose phosphate synthase (SPS, EC 2.4.1.14, BC0605), and sucrose syntase (SS, EC 2.4.1.13, BC0585) using the Solarbio kits (Solarbio, Beijing, China), the PPDK (EC 2.7.9.1, PPDK-1-Y) using the Comin kit (Suzhou, China) were measured according to the manufacturer’s instructions. Extraction of the crude enzymes occurred at 4°C. The protein concentration was measured using the Bradford kit (Coolaber, Beijing, China). Absorbance was determined with a MULTISKAN GO enzyme-labeled instrument (Thermo Fisher Scientific, USA). Three biological replicates were applied for each treatment of all samples.

CA activity was determined with method described by Brownell et al. (1991). Leaf tissues (0.1 g) were ground with 1 ml extraction solution containing 0.01 M EDTA, 0.1 M Tris-HCl (pH 8.3), and 0.05 M 1,1,1-trichloro-2,2-bis (p-chlorophenyl) ethane. The homogenate was centrifuged at 8000 g for 10 min at 4°C, and the supernatant was used as crude enzyme. The CA activity was assayed in reaction mixture containing 0.025 M 5,5-diethylbarbituric acid (pH 8.2), CO_2_-saturated water and the crude enzyme following the change in absorbance at 405 nm.

The Rubisco activity was determined by method of Camp et al. (1982). Leaf tissues (0.1 g) were ground in a mortar and pestle (4°C) in 1 ml extraction solution containing 50 mM Tricine-NaOH (pH 7.9), 0.1% polyvinylpyrrolidone (PVP), and 5 mM MgCl_2_ followed by centrifugation at 10,000 g for 10 min at 4°C, and the supernatant was used as crude enzyme. The Rubisco activity was measured in reaction mixture containing 50 mM Tricine-NaOH (pH 7.9), 10 mM KCl, 1 mM ethylene diamine tetraacetic acid (EDTA), 2 mM dithiothreitol (DTT), 0.2 mM NADH, 5 mM ATP, 15 mM MgCl_2_, 10 mM NaHCO_3_, 5 mM phosphocreatine, 2 units ml^-1^ creatine phosphokinase, 4 units ml^-1^ each of NAD-dependent glyceraldehyde 3-phosphate dehydrogenase, 3-Phosphoglycerate kinase and the crude enzyme. The absorbance change at 340 nm was recorded.

PEPC activity was measured using the method of Sayre and Kennedy (1979) with minor modifications. The functional leaves (about 0.1 g) were ground into homogenate with 1 ml of extraction solution consisting of 0.1 M Tris-HCl (pH 7.8), 10 mM MgCl_2_, 1 mM EDTA, 20 mM mercaptoethanol, 10% (w/v) glycerin and 1% PVP, followed by centrifugation at 8000 g for 20 min at 4°C, the supernatant was used as crude enzyme and added to the reaction mixture containing 50 mM Tris-HCl (pH 7.8), 10 mM MgCl_2_, 0.25 mM EDTA, 5.0 mM NaHCO_3_, 2.0 mM DTT, 4 U MDH, 0.1 mM NADH, and 2.0 mM PEP. PEPC activity was measured by following the change in absorbance at 340 nm (Zhang et al., 2008).

The activities of NADP-ME and NADP-MDH were measured by method of Yao et al. (2011) with minor modifications. The leaf tissues (0.1 g) were ground in 1 ml extraction solution containing 400 mM Tris-HCl (pH 8.2), 15 mM EDTA, 10 mM DTT, 5 mM MgCl_2_, 2% (w/v) PVP, the homogenate was centrifuged at 8000 g and 4°C for 10 min. The supernatant (crude enzyme) was added into the reaction mixture containing 50 mM Tricine-NaOH (pH 7.5), 1 mM MgCl_2_, 0.5 mM NADP, 10 mM L-malate for NADP-ME activity assay. The reaction was initiated by adding malate (Spampinato et al., 1994). For NADP-MDH activity, the reaction mixture contained 50 mM Tris-HCl (pH 7.8), 2 mM MgCl_2_, 0.5 mM EDTA, 0.2 mM NADH, 2 mM oxaloacetate (OAA) and the crude enzyme, and the absorbance change was recorded at 340 nm.

The PPDK activity was determined by the method of Sayre and Kennedy (1979). Leaf tissues (0.1 g) were homogenized in 1 ml extraction solution containing 0.1 M Tris-HCl (pH 7.8), 10 mM MgCl_2_, 1.0 mM EDTA, 20 mM mercaptoethanol and 2% (w/v) PVP. The supernatant from the centrifugation of the homogenate at 8000 g for 10 min at 4°C was used as crude enzyme and added to the reaction mixture consisting of 0.1 M Tris-HCI (pH 8.0), 10 mM MgCl_2_, 0.1 mM EDTA, 1.25 mM pyruvate, 5.0 mM DTT, 0.16 mM NADH, 2.5 mM K_2_KPO_4_, 50 mM NaHCO_3_, 2 units of corn PEP carboxylase, 3 units of malate dehydrogenase to determine the activity of PPDK. The absorbance change was recorded in a microplate reader at 340 nm.

The activities of SPS and SS were determined according to the method described by Schrader and Sauter (2002) with minor modifications. Leaf tissues (0.1 g) were ground in a mortar and pestle (4°C) in the extraction buffer containing 100 mM HEPES-NaOH (pH 7.5), 10 mM MgCl_2_, 2 mM ethylene glycoldiamin tetraacetate (EGTA), 3 mM 1,4-dithioerythritol (DTE), 2% polyethyleneglycol (PEG 8000), 0.1% Triton X-100, 0.1% bovine serum albumin (BSA), followed by centrifugation at 8000 g for 10 min at 4°C, and the supernatant as crude enzyme was added to the reaction mixture containing 0.8 mM phosphoenolpyruvate and 0.3 mM NADH as substrates,100 mM Tris-HCl (pH 7.5), 10 mM MgCl_2_ to measure the SPS activity. For SS activity, the reaction mixture contained 4 mM uridine diphosphate glucose (UDPG) and 20 mM fructose in 100 mM HEPES-NaOH (pH 7.5), 25 mM MgCl_2_. The absorbance change was recorded at 480 nm.

### 2.8 Measurement of sugar contents

The soluble sugar content was measured according to the method of Fils-Lycaon et al (2011) with minor modifications by using the Solarbio kit (Solarbio, Beijing, China). Leaf tissues (0.1 g) were ground into homogenate in 1 ml distilled water, then boiled in a water bath for 10 min, cooled down and centrifuged at 8000 g for 10 min. The supernatant was transferred into a 10 ml tube and fixed to a constant volume of 10 ml with distilled water. The reaction mixture consisting of the supernatant, distilled water, concentrated sulfuric acid and the working solution (supplied in the kit) was mixed in a water bath at 95°C for 10 min. After cooling, the absorbance changes were recorded at 620 nm. A standard curve was established according to the kit instructions.

The sucrose content was measured according to the method of Sima et al. (2001) with minor modifications by using the by using the Solarbio kit (Solarbio, Beijing, China). The leaf tissues (0.1 g) were ground into a homogenate by adding 0.5 ml of extraction buffer in a mortar and transferred to a tube in water bath at 80°C for 10 min, then centrifuged at 4000 g for 10 min after cooling. The supernatant was added again with 0.5 ml extraction buffer, centrifuged at 4000 g for 10 min, then treated with activated carbon powder in water bath at 80°C for 30 min, and then mixed with the reaction buffer in boiling water bath for 10 min according to the kit instructions, the absorbance changes were recorded at 480 nm after cooling.

### 2.9 Quantitative RT-PCR analysis

Leaf tissues of *Arabidopsis* plants were used to extract the total RNA according to the RNA kit (TIANGEN, Beijing, China) instructions. Around 2.0 μg of RNA was reverse-transcribed into cDNA using the Reverse Transcription kit (ABM, Canada). The qRT-PCR analysis was conducted with the LightCycler 96 Real-Time System (Roche, Switzerland) and ABM BlasTaq™ 2×qPCR MasterMix (ABM, Canada) according to the reaction conditions: 95°C for 2 min, followed by 40 cycles of 95°C for 15 s and 60°C for 60 s. *AtActin* was used as the internal reference, the primer sequences were presented in **Supplementary Table S1**. The 2^-ΔΔCt^ method was used to calculate the relative expression levels of genes (Livak and Schmittgen, 2001). Each treatment consisted of three biological replicates and two technical replicates.

### 2.10 Statistical analysis

Data analysis was performed using WPS Office software (Beijing, China) and SPSS 22 (IBM, New York, USA). One-way ANOVA was used to test the significance of main effects, the Tukey multiple comparison test to compare differences at a 0.05 significance level. Graphpad Prism 8 (San Diego, USA) and Origin Pro 9.1 were used to create diagrams.

## 3 Results

### 3.1 Effect of drought stress on germination and stomatal opening of different transgenic *Arabidopsis* lines

**Figure 1:**
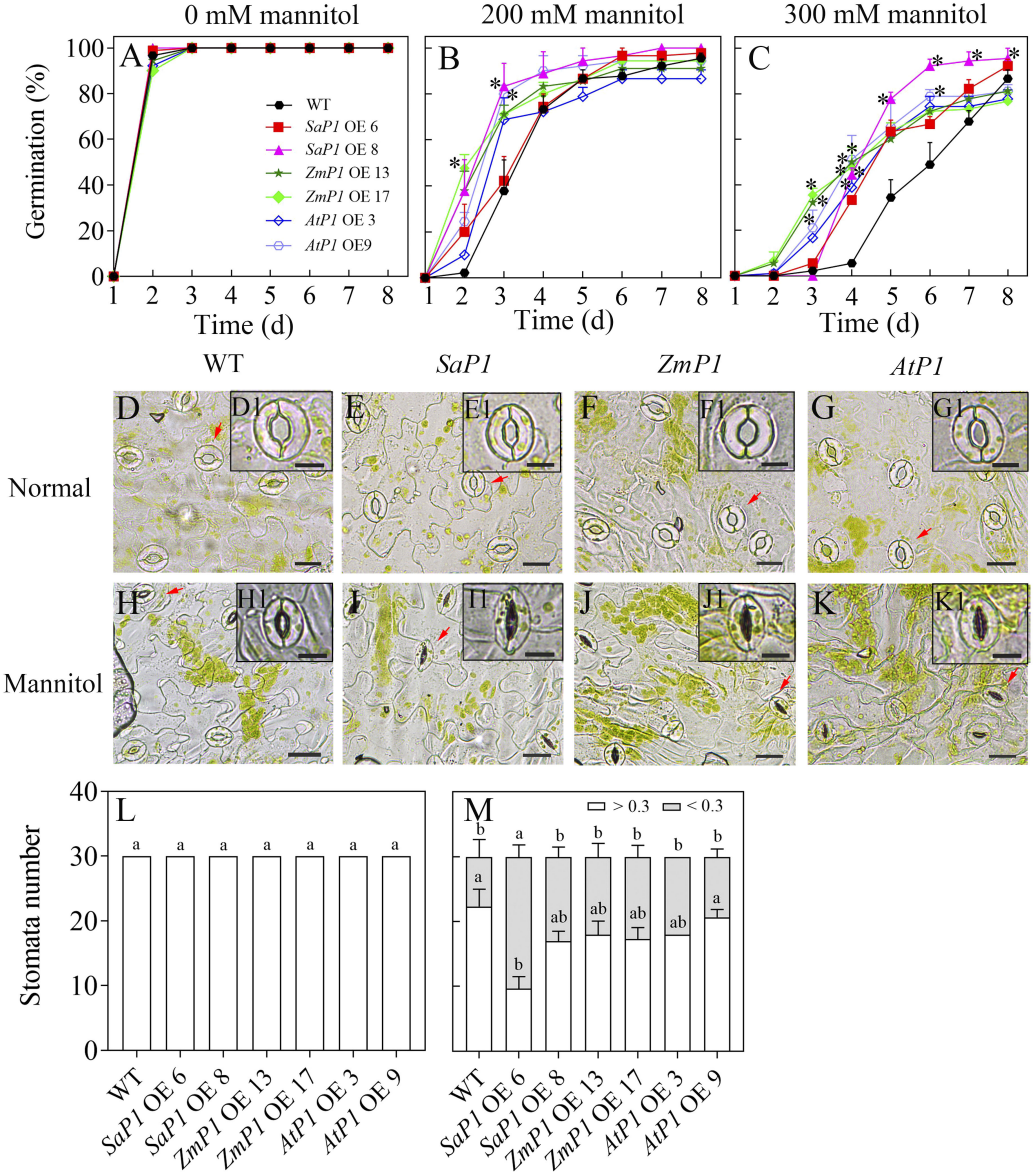
Seed germination and stomatal performance under mannitol treatment. **(A–C)** Seed germination of different transgenic *Arabidopsis* lines under 0, 200, and 300 mM mannitol treatment, respectively. **(D–G, H–K)** The stomatal morphology of different transgenic *Arabidopsis* lines under normal conditions and 300 mM mannitol treatment, respectively. WT: wild type; *SaP1* OE6, OE8: *SaPEPC1-*overexpressing transgenic lines; *ZmP1* OE13, OE17: *ZmPEPC1-*overexpressing transgenic lines; *AtP1* OE3, OE9: *AtPEPC1-*overexpressing transgenic lines. (D1–K1) The enlarged views of stoma marked with red arrows in **(D–K)**, respectively. Scale bars in **(D–K)** and D1–K1 are 20 μm and 10 μm, respectively. **(L, M)** The width-length ratio of stomata between individuals smaller and larger than 0.3 under normal conditions or 300 mM mannitol treatment, respectively. Different lowercase letters above columns represent significant differences between transgenic lines and WT under the same treatment conditions (*P* < 0.05*)*, * Indicates significant differences between WT and overexpression lines at the same time. Values are means ± SE of three replicates.

The plant expression vector was successfully constructed by enzyme digestion identification (**Supplementary Figures S1A–G**), and molecular characterization of transgenic *Arabidopsis* genotypes confirmed that *SaPEPC1*, *ZmPEPC1*, and *AtPEPC1* were integrated into the genomes of the corresponding transgenic lines (**Supplementary Figures S1H–J**). Trangenic and WT seeds germinated on medium without mannitol treatment (**Figure 1A**). The germination percentage (GP) of each transgenic line was higher than that of WT under 200 mM mannitol stress (**Figure 1B**), the difference became greater under 300 mM mannitol, and the *SaPEPC1* overexpressing *Arabidopsis* had higher GP compared to other transgenic lines (**Figure 1C**). Comparison of the stomatal open size revealed no difference between the transgenic lines and WT under normal conditions (**Figures 1D–G, L**). However, under mannitol stress, the *SaPEPC1-*6 transgenic line had significantly more stomata becoming closed compared to the WT (**Figures 1H, I, M**), the other transgenic lines also had more closed stomata but not significantly higher than the WT (**Figures 1J, K**). Our results suggest that the *PEPC* overexpressing can confer drought tolerance to *Arabidopsis* in germination and stomata activity, particularly, the *SaPEPC1* had better performance.

### 3.2 *SaPEPC1* overexpression improves antioxidant enzyme activities and reduces ROS in *Arabidopsis* under drought stress

**Figure 2:**
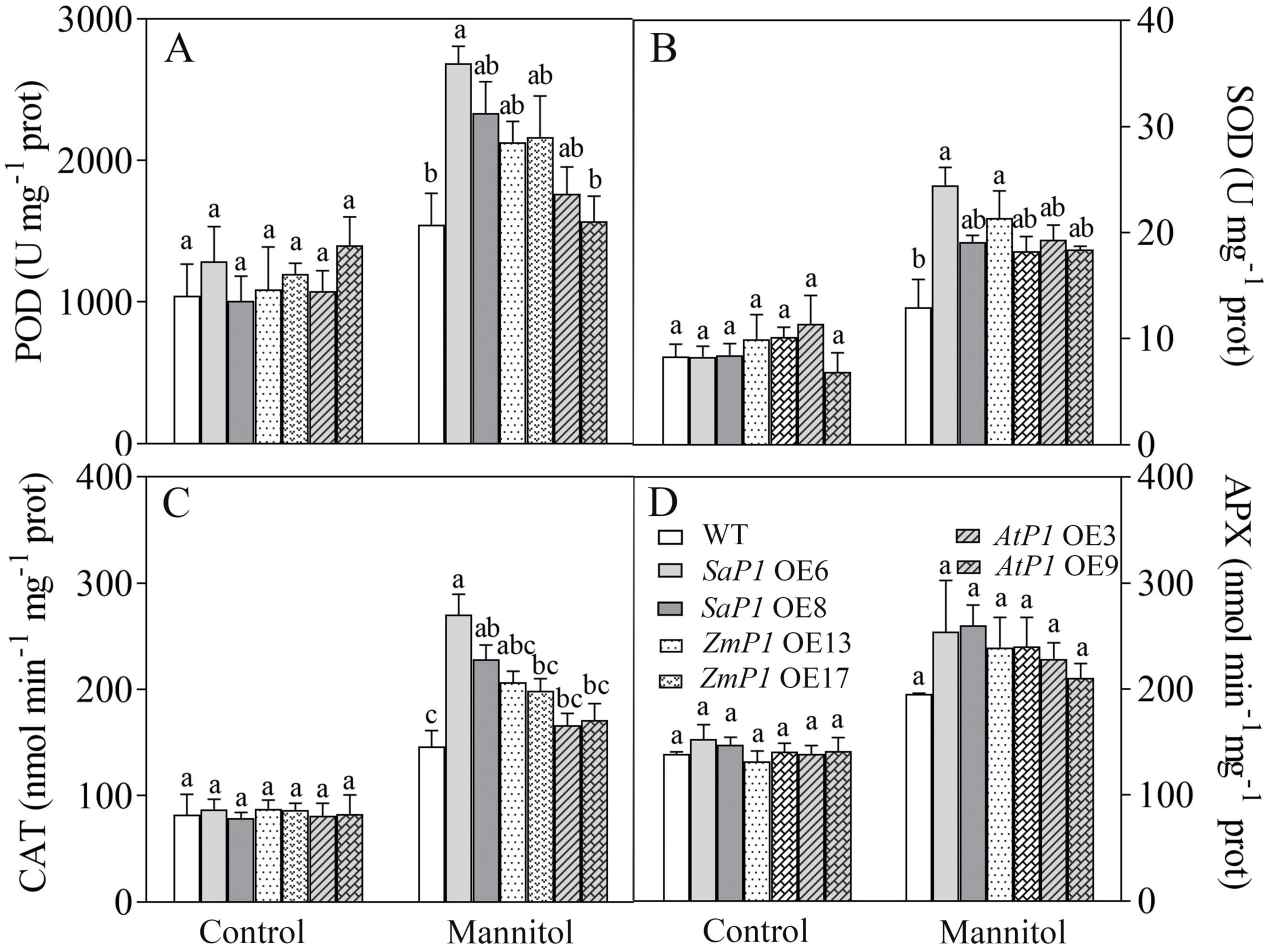
Effects of 300 mM mannitol treatment on the antioxidant enzyme activity of transgenic *Arabidopsis*. **(A–D)** The POD, SOD, CAT, and APX, respectively. WT: wild type; *SaP1* OE6, OE8: *SaPEPC1-*overexpressing transgenic lines; *ZmP1* OE13, OE17: *ZmPEPC1-*overexpressing transgenic lines; *AtP1* OE3, OE9: *AtPEPC1-*overexpressing transgenic lines. Different lowercase letters above columns indicate significant differences (*P*<0.05) between transgenic lines and WT under the same treatment. Values are means ± SE of three replicates.

**Figure 3:**
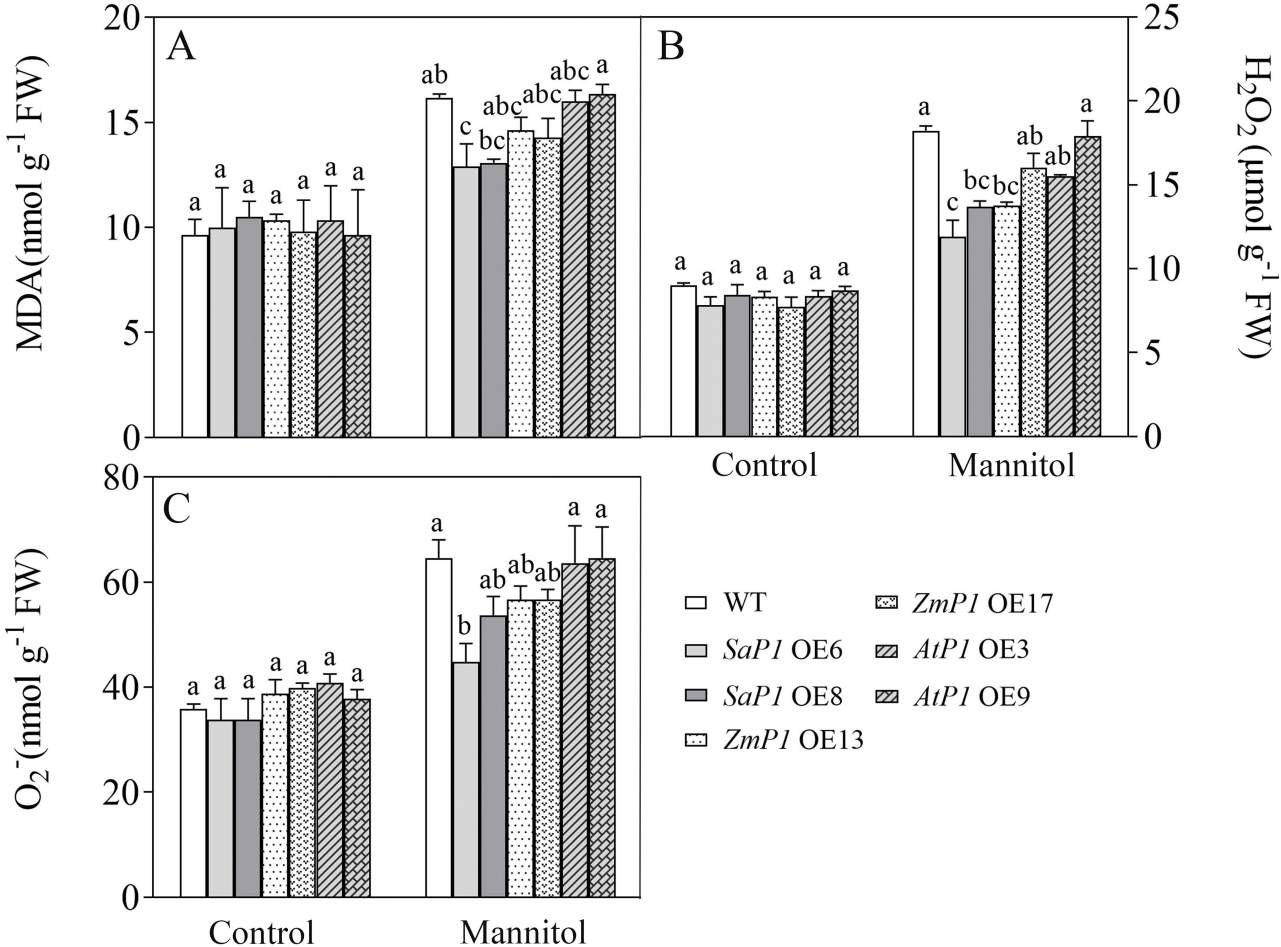
Changes of ROS levels in transgenic *Arabidopsis* under 300 mM mannitol treatment. **(A–C)** Contents of the MDA, H_2_O_2_, and O_2_^-^, respectively. WT: wild type; *SaP1* OE6, OE8: *SaPEPC1-*overexpressing transgenic lines; *ZmP1* OE13, OE17: *ZmPEPC1-*overexpressing transgenic lines; *AtP1* OE3, OE9: *AtPEPC1-*overexpressing transgenic lines. Different lowercase letters above columns indicate significant differences (*P*<0.05) between transgenic lines and WT under the same treatment. Values are means ± SE of three replicates.

There had no significantly difference in antioxidant enzyme activity (POD, SOD, CAT, APX) and ROS content between transgenic lines and WT under normal conditions, however, the POD, SOD and CAT activities of *SaPEP*C*1* and *ZmPEPC1* transgenic lines were higher than those of the WT under drought stress, particularly in *SaPEPC1*-6 line, and no significant change of antioxidant enzyme activity was detected in *AtPEPC1* transgenic lines (**Figures 2A–C**). APX activity showed no significant changes (**Figure 2D**). The increased antioxidant enzyme activity in transgenic lines correlated to the decreased ROS levels. Notably, *SaPEPC1* overexpression significantly reduced MDA and H_2_O_2_ levels (**Figures 3A, B**), while *AtPEPC1* overexpression showed similar ROS levels to WT (**Figures 3B, C**). Our results suggest that *SaPEPC1* overexpression enhances ROS scavenging ability compared to WT, whereas *AtPEPC1* overexpression had no significant effect this.

### 3.3 Analysis of photosynthetic performance of transgenic *Arabidopsis* under different light intensity

#### 3.3.1 Quick inducing the fluorescence of chlorophyll a

**Figure 4:**
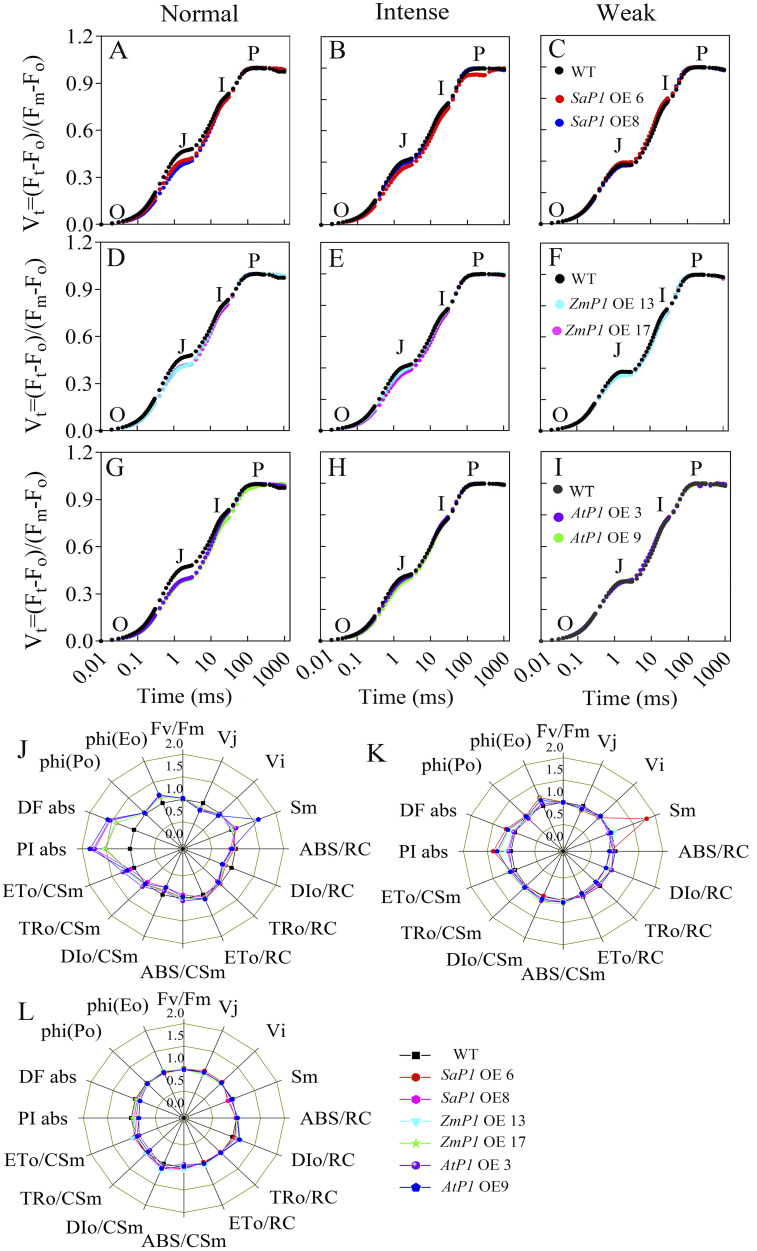
Analysis of chlorophyll fluorescence kinetic parameters in transgenic *Arabidopsis* under different light intensities. **(A–C)** Chlorophyll fluorescence kinetic curves OJIP of two *SaPEPC1-*overexpressing *Arabidopsis* lines and WT under normal, intense and weak light treatment. **(D–F)** The OJIP of two *ZmPEPC1-*overexpressing *Arabidopsis* lines and WT under normal, intense and weak light treatment. **(G–I)** The OJIP of two *AtPEPC1-*overexpressing *Arabidopsis* lines and WT under normal, intense and weak light treatment. **(J–L)** A ‘spider plot’ of selected parameters derived from the chlorophyll fluorescence OJIP curves of transgenic *Arabidopsis* lines and WT under normal, intense, and weak light treatment, J is normal light, K is intense light, L is weak light. WT: wild type; *SaP1* OE6, OE8: *SaPEPC1-*overexpressing transgenic lines; *ZmP1* OE13, OE17: *ZmPEPC1-*overexpressing transgenic lines; *AtP1* OE3, OE9: *AtPEPC1-*overexpressing transgenic lines. All data of JIP test parameters were normalized to the reference WT. Values are means ± SE of six replicates.

Drought stress closely correlates with the photosynthesis cycle in terrestrial ecosystems (Sekhar et al., 2021). Since light significantly promotes *PEPC* transcripts accumulation in leaves (Tolley et al., 2012), we investigated the impact of *PEPC* on photosynthetic performance under varying light intensities. In the OJIP (the rapid chlorophyll fluorescence induction kinetics) curve, the O-J stage of the transgenic *Arabidopsis* was notably decreased compared with WT under normal light conditions (**Figures 4A, D, G**). Under intense light, the O-J and O-I stages of *SaPEPC1* or *ZmPEPC1* transgenic *Arabidopsis* were slightly lower than WT (**Figures 4B, E**), whereas *AtPEPC1* lines showed no difference (**Figure 4H**). Under weak light, the OJIP curves of transgenic *Arabidopsis* and WT exhibited similar trends (**Figures 4C, F, I**). JIP-test analysis revealed smaller DIo/RC and DIo/CSm in transgenic lines than WT. In contrast, transgenic plants had higher ETo/CSm, PIabs, DFabs, and phi(Eo) values (**Figure 4J**). Notably, *SaPEPC1*-6 line had a significantly larger Sm under intense light, with slightly larger PIabs, DFabs, and phi(Eo) than WT (**Figure 4K**). However, under weak light, DFabs and PIabs were smaller in transgenic plants than WT (**Figure 4L**). OJIP curve and JIP-test parameters indicated that transgenic plants dissipated less energy per unit reaction center under normal light, exhibiting better PSII performance. Particularly, the quinone electron acceptor (Q_A_) of *SaPEPC1*-6 line presented robust energy conservation ability and performance index from PSII-absorbed photons until intersystem electron acceptor reduction under intense light. No significant difference in the primary reaction was observed between transgenic *Arabidopsis* and WT under weak light.

#### 3.3.2 Relative electron transfer rate

**Figure 5:**
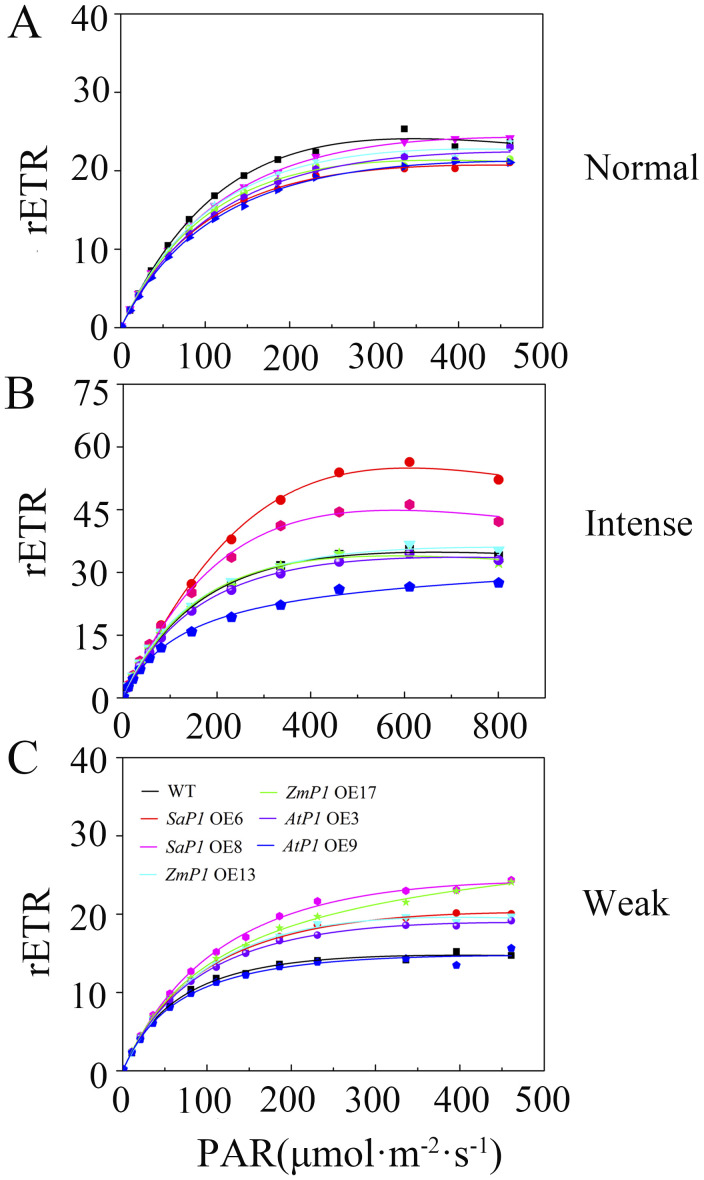
Relative electron transfer rate (rETR) of transgenic *Arabidopsis* under different light intensities. **(A)** Normal light. **(B)** Intense Light. **(C)** Weak Light. WT: wild type; *SaP1* OE6, OE8: *SaPEPC1-*overexpressing transgenic lines; *ZmP1* OE13, OE17: *ZmPEPC1-*overexpressing transgenic lines; *AtP1* OE3, OE9: *AtPEPC1-*overexpressing transgenic lines. Values are means ± SE of six replicates.

Under normal light conditions, the transgenic *Arabidopsis* line’s rETR was slightly lower than the WT, and reached maximum stability at about 250 μmol·m^-2^·s^-1^ PAR (**Figure 5A**). However, under intense light conditions, the rETR value of the *SaPEPC1* transgenic *Arabidopsis* was higher than that of the WT, with *SaPEPC1*-6 and line *SaPEPC1*-8 lines reaching maximum values of 56.38 and 46.25, respectively. At approximately 461 μmol·m^-2^·s^-1^ PAR, the rETR value stabilized, but the maximum rETR value of *AtPEPC1*-9 line was 27.48, which was significantly lower than that of the WT (**Figure 5B**). In weak light conditions, both the *AtPEPC1* transgenic *Arabidopsis* and WT stabilized at about 200 μmol·m^-2^·s^-1^ PAR, with the rETR values of *AtPEPC1* transgenic *Arabidopsis* and WT tending to be stable. While *AtPEPC1*-9 line was similar to that of the WT, other transgenic lines stabilized at obviously higher values than the WT at around 250 μmol·m^-2^·s^-1^ PAR (**Figure 5C**). These findings suggest that *SaPEPC1* overexpression not only enhances *Arabidopsis*’s tolerance to intense light, but also inproved photosynthetic capacity under intense light, whereas *AtPEPC1* transgenic *Arabidopsis* can not significantly improve *Arabidopsis*’s photosynthetic performance.

#### 3.3.3 Chlorophyll fluorescence parameters

Under normal light conditions, there was little difference in the maximum quantum efficiency (yield) of PSII photochemistry (Fv/Fm), PSII actual quantum yield [Y(II)], non-regulatory energy dissipation [Y(NO)], non-photochemical quenching (qN), and photochemical quenching (qP) between transgenic plants and the WT (**Supplementary Figures S2A, D, G, J, M**). However, under intense light treatment, *SaPEPC1* overexpression significantly elevated Y(II) and qP compared to WT (**Supplementary Figures S2E, N**). Conversely, no significant difference in fluorescence parameters was observed between transgenic lines overexpressing *ZmPEPC1* and WT (**Supplementary Figures S2B, E, H, K, N**), and although the Y(II) of *AtPEPC1* transgenic *Arabidopsis* was lower than that of WT, but did not attain statistical significance (**Supplementary Figures S2B, E**). Under weak light conditions, overexpression of *ZmPEPC1* significantly boosted Fv/Fm in comparison to WT (**Supplementary Figure S2C**), while overexpression of *SaPEPC1* increased Y(II) and Y(NO) relative to WT (**Supplementary Figures S2F, I**), with a simultaneously significant reduction in qN compared to WT (**Supplementary Figure S2L**). However, no significant differences in qP were observed between transgenic lines and WT (**Supplementary Figure S2O**). These findings suggest that overexpression of *SaPEPC1* can enhance light energy conversion efficiency and facilitates timely dissipation of excess light energy to mitigate potential damage from light exposure.

#### 3.3.4 Gas exchange parameter

**Figure 6:**
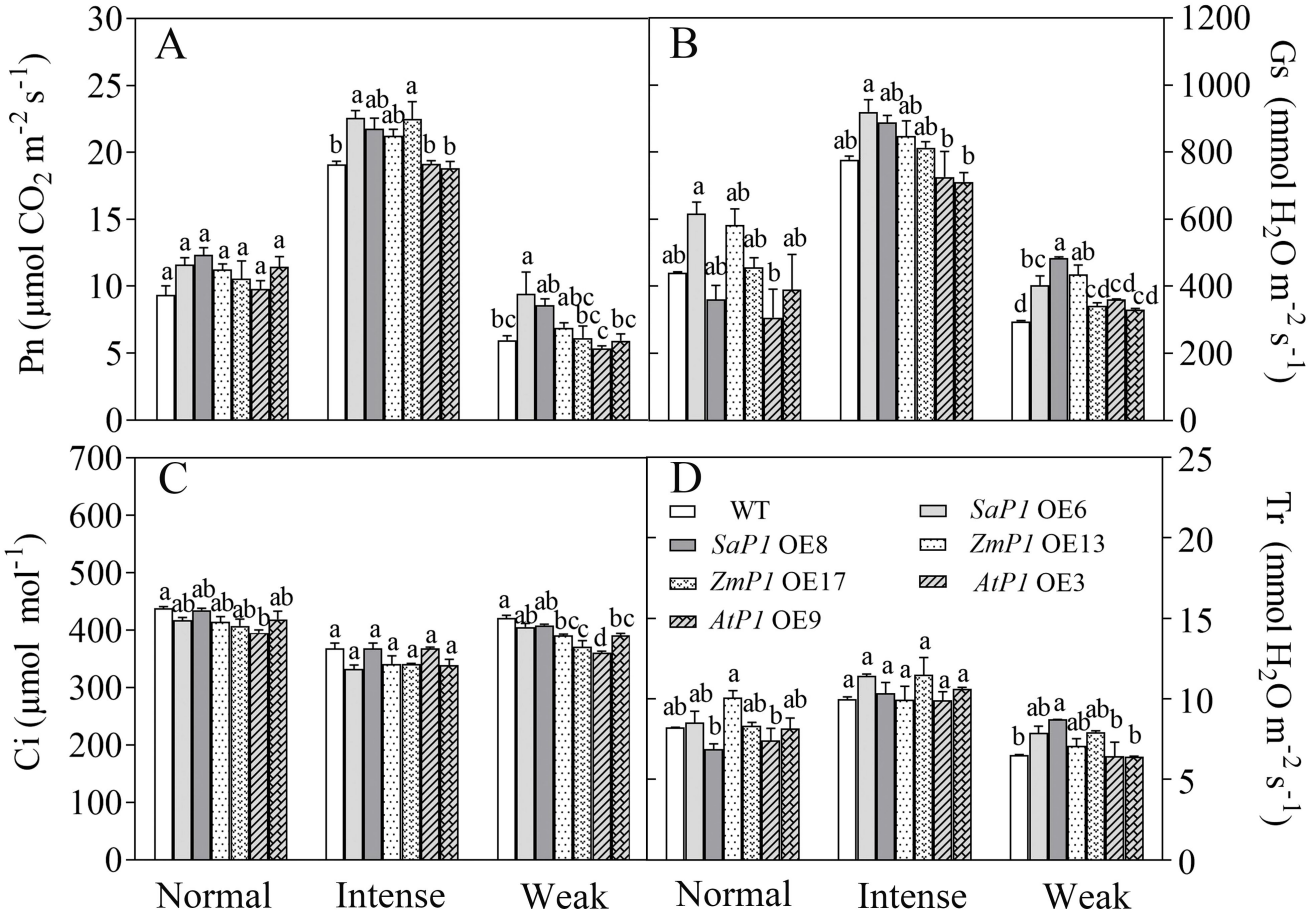
Changes in gas exchange parameters of transgenic *Arabidopsis* under different light intensity treatments. **(A)** Net photosynthetic rate (Pn). **(B)** Stomatal conductance (Gs). **(C)** Intercellular CO_2_ concentration (Ci). **(D)** Transpiration (Tr). WT: wild type; *SaP1* OE6, OE8: *SaPEPC1-*overexpressing transgenic lines; *ZmP1* OE13, OE17: *ZmPEPC1-*overexpressing transgenic lines; *AtP1* OE3, OE9: *AtPEPC1-*overexpressing transgenic lines. Different lowercase letters above columns represent significant differences between transgenic lines and WT under the same treatment (*P* < 0.05). Values are means ± SE of three replicates.

Under different light intensity treatments, transgenic *Arabidopsis* lines and WT had the highest Pn, Gs and Tr under intense light, followed by normal light, and the lowest in the weak light, while Ci was lower in the intense light than in the normal light and weak light treatments. Under intense light treatment, The Pn of *SaPEPC1* OE6, OE8 increased by 18.15%, 13.96% (**Figure 6A**), Gs increased by 18.50%, 14.42% (**Figure 6B**), Tr increased by 14.61%, 3.81% (**Figure 6D**), and Ci was 9.77%, 3.08% lower than that of WT (**Figure 6C**). While the Pn of *ZmPEPC1* transgenic lines under intense light was extremely close to *SaPEPC1*, Gs was lower than *SaPEPC1*, however, overexpression of *AtPEPC1* had no significant effect on Pn, Gs, Ci and Tr under different light intensity treatments. These results indicate that the overexpression of *SaPEPC1* or *ZmPEPC1* can enhance the photosynthetic efficiency and promote gas exchange of *Arabidopsis*.

### 3.4 Effect of light intensity on photosynthetic enzyme activity, sugar content and related enzyme activities of different transgenic *Arabidopsis* lines

#### 3.4.1 Photosynthetic enzyme activity

**Figure 7:**
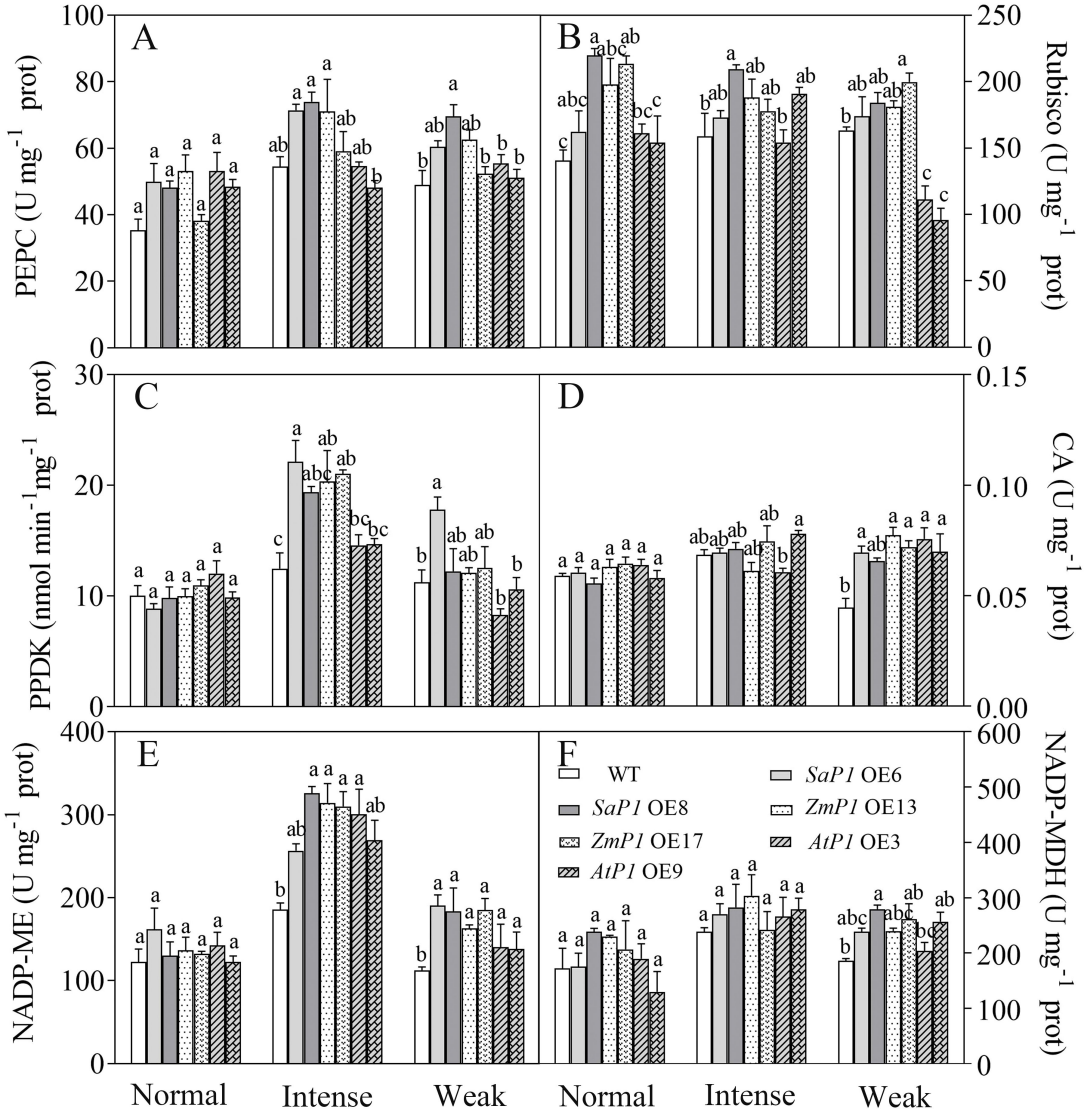
Analysis of photosynthetic enzyme activities in transgenic *Arabidopsis* under different light intensities. **(A)** PEPC. **(B)** Rubisco. **(C)** PPDK. **(D)** CA. **(E)** NADP-ME. **(F)** NADP-MDH. WT: wild type; *SaP1* OE6, OE8: *SaPEPC1-*overexpressing transgenic lines; *ZmP1* OE13, OE17: *ZmPEPC1-*overexpressing transgenic lines; *AtP1* OE3, OE9: *AtPEPC1-*overexpressing transgenic lines. Different lowercase letters above columns indicate significant differences between transgenic lines and WT under the same treatment (*P* < 0.05). Values are means ± SE of three replicates.

Under normal light, the enzyme activity of PEPC and Rubisco in transgenic lines was higher than that of WT. However, under intense light, the activities of PEPC, PPDK, and NADP-ME in *SaPEPC1* or *ZmPEPC1* transgenic *Arabidopsis* were higher compared to both the WT and the transgenic lines grown under normal light. Specifically, the PEPC activity in *SaPEPC1* OE6, OE8 two transgenic lines was 31.04% and 35.77% higher than WT, PPDK activity was 77.25% and 55.29% higher than WT, and NADP-ME activity was 37.99% and 75.31% higher than WT, respectively (**Figures 7A, C, E**). Conversely, the enzyme activities of Rubisco and NADP-MDH exhibited minimal changes under intense light (**Figures 7B, F**). Under weak light conditions, the Rubisco activity in *AtPEPC1* transgenic line was notably lower than that of the WT and other transgenic lines (**Figure 7B**). However, there were no significant changes in the CA enzyme activity between the transgenic and WT plants under different light intensity treatments (**Figure 7D**). This suggests that intense light promotes the activity of key enzymes of C4 photosynthesis including PEPC, PPDK and NADP-ME in *Arabidopsis* overexpressing *SaPEPC1* and *ZmPEPC1* genes.

#### 3.4.2 Sugar content and related enzyme activity

**Figure 8:**
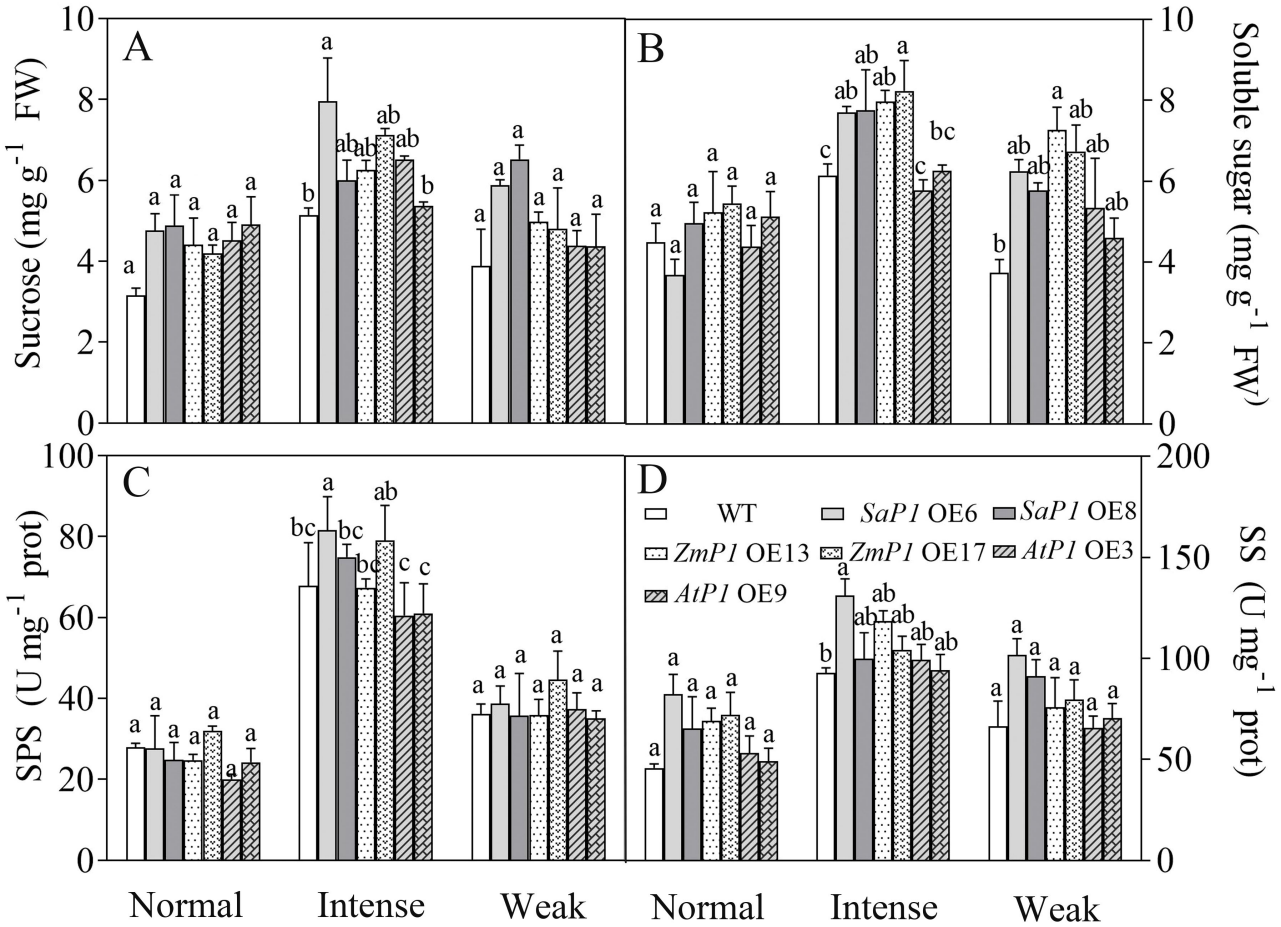
Effects of different light intensity treatments on sugar content and sugar synthesis-related enzyme activities in transgenic Arabidopsis. **(A)** Sucrose. **(B)** Soluble sugar. **(C)** SPS. **(D)** SS. WT: wild type; *SaP1* OE6, OE8: *SaPEPC1-*overexpressing transgenic lines; *ZmP1* OE13, OE17: *ZmPEPC1-*overexpressing transgenic lines; *AtP1* OE3, OE9: *AtPEPC1-*overexpressing transgenic lines. Different lowercase letters above columns indicate significant differences between transgenic lines and WT under the same treatment (*P* < 0.05). Values are means ± SE of three replicates.

Under normal and weak light conditions, there were no significant differences in the sucrose content, SPS and SS enzyme activities between transgenic line and WT plants. However, under intense light conditions, *SaPEPC1* or *ZmPEPC1* transgenic *Arabidopsis* exhibited higher sucrose and soluble sugar contents compared with WT, and the soluble sugars of them reached significant differences (**Figures 8A, B**). The SPS and SS enzyme activities of *SaPEPC1* OE6 and OE8 transgenic lines were higher than those of WT, and OE6 reached significant difference (**Figures 8C, D**). Moreover, the soluble sugar content of transgenic plants surpassed that of WT under weak light conditions (**Figure 8B**). These findings suggest that overexpression of *SaPEPC1* or *ZmPEPC1* promotes sucrose accumulation under intense light.

### 3.5 Transcriptional levels of the photosynthesis and sugar synthesis-related genes in transgenic *Arabidopsis*

**Figure 9:**
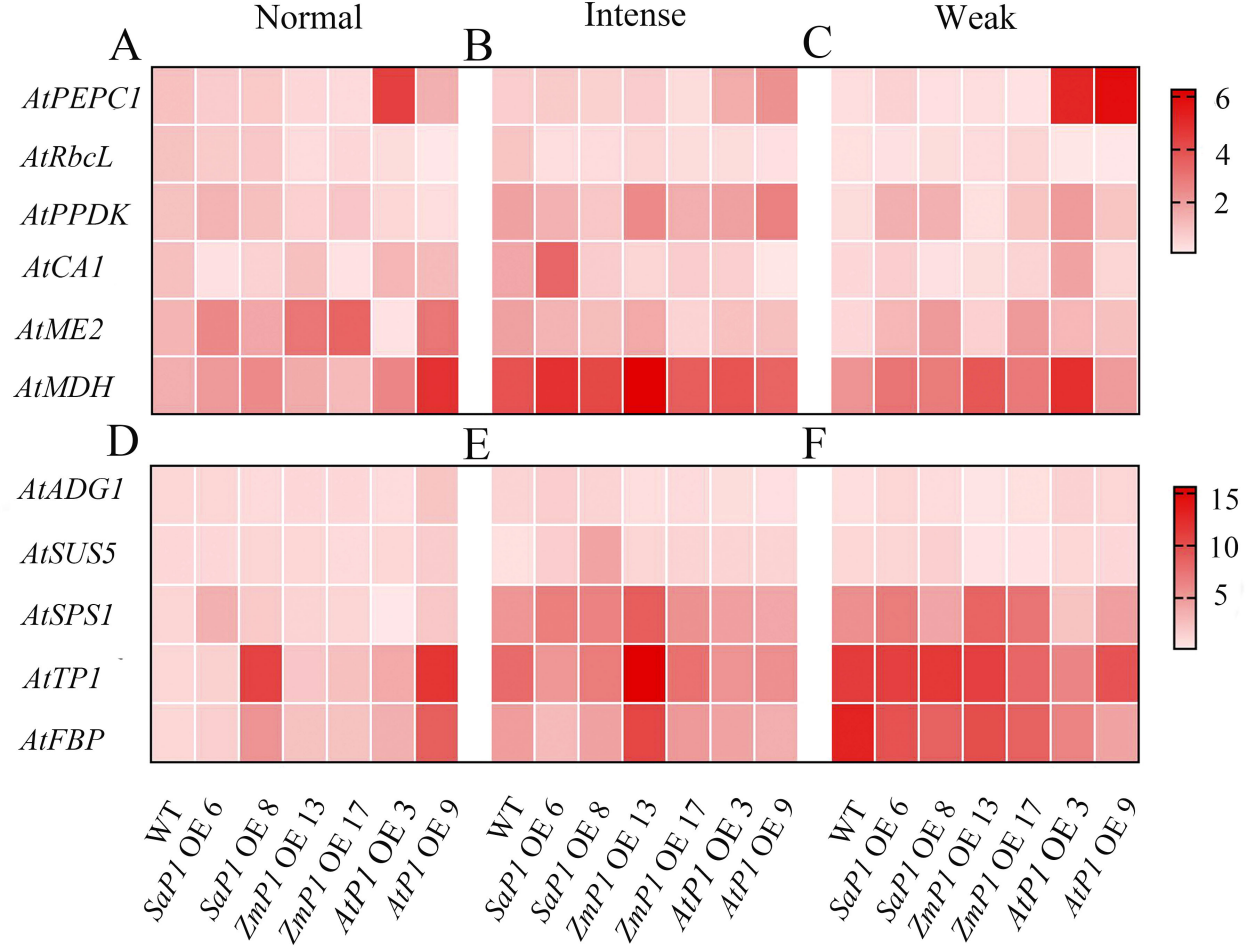
qRT-PCR analysis of the transcriptional expression pattern of key photosynthesis and sugar synthesis-related genes in the transgenic *Arabidopsis*. **(A–C)** Photosynthesis-related genes under normal, intense and weak light, respectively. **(D–F)** Suger synthesis-related genes under normal, intense and weak light. WT: wild type; *SaP1* OE6, OE8: *SaPEPC1-*overexpressing transgenic lines; *ZmP1* OE13, OE17: *ZmPEPC1-*overexpressing transgenic lines; *AtP1* OE3, OE9: *AtPEPC1-*overexpressing transgenic lines. Values are means ± SE of three biological and two technical replicates (total of six replicates).

The expression of photosynthesis-related genes presented more significant changes under weak light compared to the normal and intense light conditions (**Figures 9A–C**). Specifically, the transcript accumulation of *SaPEPC1* or *ZmPEPC1* transgenic *Arabidopsis* was lower under weak light than that under normal and intense light conditions. Similarly, the expression level of *AtRbcL* in both transgenic and WT plants was lower under weak light compared to normal and intense light conditions overall. Notably, transgenic lines overexpressing *AtPEPC1* exhibited lower *AtRbcL* expression level (**Figures 9C, F**). In *SaPEPC1* OE6 under intense light, the expression level of *AtCA1* was much higher than in WT, while in all other transgenic lines, it was lower than the WT. *Arabidopsis* overexpressing *SaPEPC1* showed higher expression levels of *AtPPDK*, *AtME2*, and *AtMDH* compared to the WT (**Figure 9B**). The changes in *AtADG1* expression were not evident among transgenic lines under different light treatments (**Figures 9D–F**). Under intense light conditions, the expression level of *AtSUS5* and *AtSPS1* in *SaPEPC1* transgenic lines was higher than in WT. In addition, the expression level of *AtSPS1*, *AtTPI*, and *AtFBP* in *ZmPEPC1* OE13 was significantly higher than in other transgenic lines. Conversely, the expression level of *AtSPS1*, *AtTPI*, and *AtFBP* in *AtPEPC1* transgenic *Arabidopsis* was lower than in WT (**Figure 9E**).

## 4 Discussion

Numerous studies have demonstrated the pivotal role of *PEPC* family genes in regulating plant photosynthesis and abiotic stress (Zhao et al., 2019; Chen et al., 2023). In this investigation, we found that the overexpression of *SaPEPC1* or *ZmPEPC1* enhanced drought resistance in transgenic *Arabidopsis* by inducing stomatal closure, boosting antioxidant enzyme activity, and mitigating ROS accumulation to preserve membrane stability. Additionally, under intense light conditions, overexpressed *SaPEPC1* or *ZmPEPC1* enhanced electron transport efficiency, PSII photochemical efficiency, and the activity of key C4 photosynthetic enzymes such as PEPC, PPDK, and NADP-ME. This augmentation facilitates better light energy capture, dissipation of excess light energy, improved photosynthetic efficiency, and increased accumulation of sucrose and soluble sugars. However, overexpression of *SaPEPC1* enhanced *Arabidopsis* electron transport rate, PSII actual quantum yield and photoprotective ability significantly more than overexpression of *ZmPEPC1.* Moreover, weak light conditions upregulate the expression of photosynthetic-related genes in transgenic lines, although other indices remain largely unaffected compared to intense light conditions. Notably, overexpression of *AtPEPC1* did not exert a significant effect on the drought resistance or photosynthetic performance of *Arabidopsis*. These findings suggest the potential utility of *SaPEPC1* as a promising candidate gene for transgenic breeding aimed at enhancing the photosynthetic efficiency and drought resistance of C3 plants.

Drought stress, a prevalent abiotic stressor, induces degradation of the photosynthetic apparatus, dysregulation of metabolic pathways, alteration of cellular osmotic balance, and disruption of the delicate ROS production-scavenging equilibrium (Mathur and Roy, 2021). Additionally, it diminishes both the content and activity of photosynthetic carbon-cycle enzymes (Reddy et al., 2004). C4 plants exhibit superior drought tolerance compared to C3 plants due to the ability of PEPC, a primary carboxylase, to immobilize low intercellular CO_2_, thereby facilitating organic matter accumulation. Conversely, photosynthesis in C3 plants is severely hampered under conditions of extremely low intercellular CO_2_ (Bandyopadhyay et al., 2007). Studies have demonstrated that the overexpression of *ZmPEPC* in rice enhances drought tolerance, augments antioxidant defense and yield, and modulates sugar metabolism (Zhang et al., 2017). Our investigation revealed that the overexpression of *SaPEPC1* or *ZmPEPC1* in transgenic *Arabidopsis* increased antioxidant enzyme activities and reduced ROS levels under drought stress, whereas no discernible difference was observed between *AtPEPC1-*overexpressed *Arabidopsis* and the WT. These results indicate that the drought resistance conferred by the *AtPEPC1* gene from C3 plants is inferior to that of the *PEPC* gene from C4 plants such as maize and *S. aralocaspica*. Stomatal regulation constitutes a pivotal mechanism enabling plants to withstand drought stress and adapt to their environment. The initial response of plants to drought stress signals is stomatal closure, thereby restricting gas exchange between the atmosphere and leaf interiors (Saglam et al., 2014). Our findings indicate that transgenic plants exhibited a higher frequency of stomatal closure under drought stress compared to the WT, coupled with a superior seed germination rate, suggesting that *PEPC* may mitigate water loss by facilitating stomatal closure, consequently enhancing drought resistance (Outlaw et al., 2002).

Chlorophyll fluorescence serves as a direct, non-destructive monitoring method for assessing plant photosynthetic performance (Baker, 2008; Ni et al., 2019). It encompasses the primary light reaction, electron transport, and light energy utilization (Kalisz et al., 2023). The OJIP curve allows continuous recording of fluorescence signals ranging from minimum fluorescence (O) to maximum fluorescence (P), commonly employed in investigating the activity and electron transport of PSII reaction centers (Yusuf et al., 2010; Mathur et al., 2021). The J step denotes the fluorescence value change after a 2 ms reaction, indicative electron transfer of PSII Q_A_ (Haldimann and Strasser, 1999). In this study, under normal light conditions, the O-J step of transgenic *Arabidopsis* appeared lower than that of WT, suggesting that the *PEPC* gene potentially enhances PSII reaction center activity and Q_A_ electron transmission rate. Fluctuations in natural light intensity pose challenges to plant growth, with OJIP tests reflecting the intrinsic PSII efficiency of plants (Benkov et al., 2019). *ZmPEPC*’s significance in maintaining robust photosynthetic performance under intense light in transgenic *Arabidopsis* has been established in studies, e.g., enhancing PSII activity, electron transfer capacity, and photochemical efficiency (Zhang et al., 2021). In our study, we observed that intense light upregulated *SaPEPC1* and *ZmPEPC1*, with the O-J step slightly lower than WT; whereas *AtPEPC1*’s OJIP curve was similar to the WT, suggesting heightened light tolerance in *SaPEPC1* and *ZmPEPC1* transgenic *Arabidopsis* compared to *AtPEPC1*. Moreover, in *SaPEPC1* OE6, the Sm significantly surpassed WT level, indicating Q_A_’s superior electron transfer capability under intense light. Conversely, transgenic *Arabidopsis* exhibited WT-like OJIP curves under weak light, albeit with reduced DFabs and PIabs, indicating reduced primary photosynthetic reaction under such conditions.

Plant leaves absorb light energy in three ways. Most of this energy is utilized for photosynthetic electron transport. Excess light energy is dissipated as heat, while some exists as chlorophyll fluorescence (White and Critchley, 1999). The light response curve tracks changes in ETR with varying light intensity. ETR increases rapidly until it stabilizes at light saturation, with higher ETR values indicating greater photosynthetic capacity (Liran et al., 2020; Figueroa et al., 2003). Studies have demonstrated that overexpressed *PEPC* enhances *Arabidopsis* ETR and reduces the nonphotochemical burst coefficient (Kandoi et al., 2016). Our study found that overexpressed *SaPEPC1* significantly boosted *Arabidopsis*’ photosynthetic capacity, with rETR values much higher than WT under intense light. Fv/Fm is an important indicator of photoinhibition degree (Maxwell and Johnson, 2000), which was not observed under the light intensity set in this study. Furthermore, the light saturation photosynthetic rate and carboxylation efficiency of the *ZmPEPC* gene in rice increased by 55% and 50%, respectively, compared to non-transgenic rice (Jiao et al., 2001). Y(II) indicates the energy portion utilized for a photochemical reaction post-PSII absorption, reflecting the actual photosynthetic rate (Xu et al., 2020). Among the tested enzymes, *SaPEPC1* overexpression yielded the highest Y(II) value, followed by *ZmPEPC1* overexpression, with *AtPEPC1* overexpression demonstrating the lowest value under both intense and weak light conditions. These findings underscore the significant contribution of *SaPEPC1* to *Arabidopsis*’ actual photosynthetic rate. In bright sunlight, plants employ a non-photochemical quenching mechanism to dissipate excess light energy, preventing ROS accumulation that can damage photosynthetic structure (De Souza et al., 2022). In this study, overexpression of *SaPEPC1* resulted in higher qP and lower qN compared to other enzymes under intense light, while Y(NO) showed negligible variation between transgenic *Arabidopsis* and WT, suggesting enhanced photoprotective capabilities due to *SaPEPC1* overexpression, and the absorbed light energy is appropriately divided between photochemical electron transport and heat dissipation (Wang et al., 2017).

C4 photosynthesis enzymes enhance plant water utilization and are crucial for photosynthetic efficiency. Notably, these enzymes are not exclusive to C4 plants but are also present in C3 plants (Hibberd and Quick, 2002). Rice overexpressing *ZmPEPC* was significantly increased in PEPC enzyme activity compared to WT, while no disparity was observed in Rubisco activity between the two (Jiao et al., 2001). This finding aligns with the outcomes of our study. Häusler et al. (2001) demonstrated that overexpression of *StPEPC* in potatoes led to a 3-6 fold augmentation in endogenous enzyme activities such as NADP-ME, PPDK, PEPC, and NADP-GADPH. Our study confirms this observation, indicating a similar trend in response to intense light treatment, correlating with photosynthetic performance outcomes. C4 photosynthetic gene expression is influenced by light and metabolic signals (Kausch et al., 2001). Maize C4-PEPC expression has been shown to be organ-specific and light-dependent (Ku et al., 1996). The PPDK enzyme activity in transgenic rice leaves is regulated by light as in C4 plants (Taniguchi et al., 2008). In the facultative C4 NADP-ME type plant *Hydrilla verticillata*, the transcription of the C4 type *HvPEPC4* was substantially upregulated under light and subjected to post-translational phosphorylation under light. The PEPC activity of C4 leaves was higher and showed allosteric regulation (Rao et al., 2006). In our study, the C4-type *PEPC* overexpression presented a large change in PEPC activity, whereas the C3-type *PEPC* overexpression showed a minimal change in PEPC activity. This may be related to the fact that PEPC is regulated not only at the transcriptional and post-translational level but also by metabolites (Taybi et al., 2004; Jiao and Chollet, 1991). Additionally, overexpression of *ZmPEPC* in wheat promotes the expression of endogenous photosynthetic genes including *TaPEPCK*, *TaCA*, *TaNADP-MDH*, *TaFBP* and *TaTPT* (Qi et al., 2017). Contrarily, we observed no significant enhancement in the expression of photosynthetic genes in transgenic *Arabidopsis* under intense light. However, *Arabidopsis* overexpressing *SaPEPC1* exhibited higher levels of *AtPPDK*, *AtME2*, and *AtMDH* compared to WT under weak light conditions. Previous studies have indicated that PEPC enzyme activity is influenced by various factors such as cytoplasmic pH, post-translational regulation, phosphorylation, and substrate concentration (Jeanneau et al., 2002; Schäffner and Sheen, 1992). Thus, the regulation of enzyme activity involves complex mechanisms, and the disparity between gene expression and enzyme activity warrants further investigation.

Terrestrial plants rely on effective enhancement and distribution of photosynthetic products (He et al., 2020). PEPC plays a crucial role in bolstering cassava’s drought tolerance by facilitating ample CO_2_ provision for Rubisco’s carbon sequestration, consequently elevating sucrose yield in cassava leaves (Punyasu et al., 2023). In this study, transgenic *Arabidopsis* exhibited no significant changes in sucrose levels, soluble sugar content, or SPS and SS enzyme activities under normal light conditions compared to WT. However, the rice plants overexpressing *ZmPEPC* showed significant increases in soluble sugar content, SPS and SS enzyme activities during the flowering stage (Li and Wang, 2013). This discrepancy may stem from variations in plant developmental stages, influencing sugar accumulation and enzyme activities. Conversely, exposure to intense light effectively stimulated sugar accumulation and enzyme activity in *Arabidopsis* overexpressing *SaPEPC1* or *ZmPEPC1*. Concurrently, intense light upregulated the expression of *AtSUS5* and *SPS1* genes associated with sugar synthesis. This suggests that *PEPC* induction by intense light triggers the upregulation of sugar synthesis-related genes, thereby facilitating sugar synthesis. Therefore, we speculated that *SaPEPC1* is induced by intense light, thus significantly enhancing the electron transfer rate, PSII actual quantum yield and photosynthetic rate of transgenic *Arabidopsis* under intense light, and at the same time promoting the increase of PEPC, PPDK, NADP-ME and other key enzyme activities in transgenic *Arabidopsi*s, which may generate a C4-like microcirculation in cytoplasm of *Arabidopsis*, thus enhancing the CO_2_ concentration function (Taniguchi et al., 2008), resulting in an increase in photosynthetic products (**Figure 9**). However, the changes in metabolism mechanism of C3 plants caused by overexpression of C4 type PEPC still need to be further studied.

## 5 Conclusion

**Figure 10:**
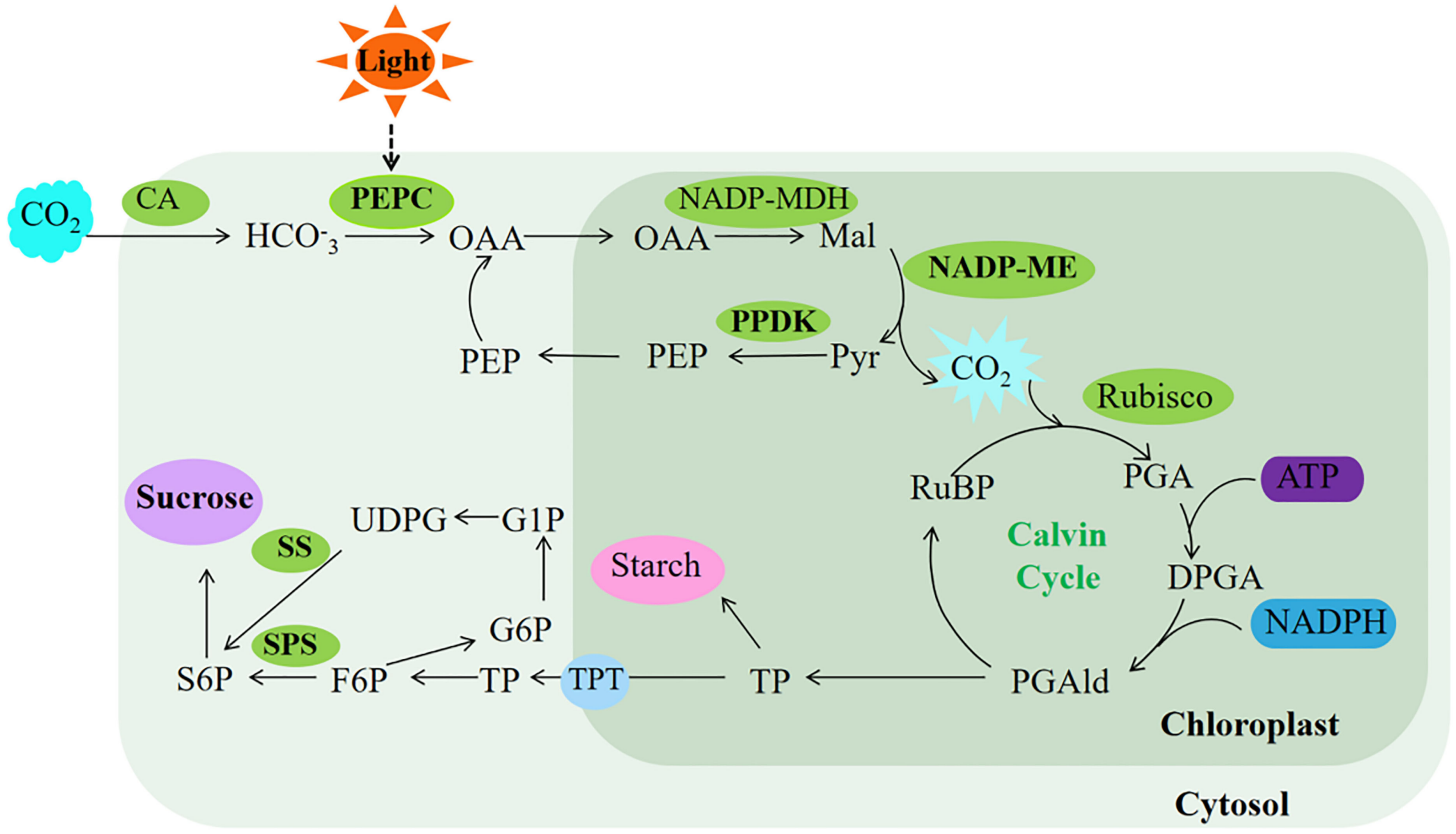
Proposed model of photosynthesis in transgenic *Arabidopsis.* The ellipses in the limegreen background represent the enzyme activity measured in this study, and the ellipses in the purple background represent the major photosynthetic products of Arabidopsis. The light green background represents photosynthetic reactions completed in the cytosol, while the dark green represents photosynthetic reactions completed in the chloroplast. OAA, oxaloacetate; Mal, malate; Pyr, pyruvate; PEP, phosphoenolpyruvate; RuBP, ribulose-1,5-bisphosphate; PGA, 3-phosphoglycerate; DPGA, 3-diphosphoglyceric; PGAld, 3-phosphoglyceraldehyde; TP, triose phosphate; TPT, triose phosphate translocator; F6P, fructose-6-phosphate; S6P, sucrose 6-phosphate; G6P, glucose 6-phosphate; G1P, glucose 1-phosphate; UDPG, UDP-glucose. The bold font represent substances upregulated under intense light treatment in this study.

Our data indicated that introducing *PEPC*, a key gene in the C4 photosynthetic cycle, into C3 plants significantly enhances their drought resistance. Specifically, overexpression of *PEPC* favors the primary photosynthetic reaction under normal light conditions, albeit less conspicuously in weak light. Moreover, overexpressed *SaPEPC1* can significantly improve photosynthetic efficiency and photoprotection capabilities. This enhancement in photosynthetic capacity arises not only from increased activity of various key photosynthetic enzymes but also potentially from the CO_2_ concentration mechanism inherent in the C4-like cycle pathway, resulting in sucrose production (**Figure 10**). Our study offers novel insights into *SaPEPC1*-driven photosynthesis and identifies candidate genes for enhancing photosynthetic performance in C3 plants. However, unraveling the intricate mechanisms and disparities between *SaPEPC1* with single-cell C4 cycle and *ZmPEPC1* with Kranz anatomical structure in drought resistance and photosynthesis poses a considerable challenge. Thus, further research is imperative for a comprehensive understanding in the future.

## Data Availability

The original contributions presented in the study are included in the article/**Supplementary Material**. Further inquiries can be directed to the corresponding authors.
