# Supplementary material for: Enhanced drought tolerance and photosynthetic efficiency in Arabidopsis by overexpressing phosphoenolpyruvate carboxylase from a single-cell C4 halophyte Suaeda aralocaspica
Source: Front Plant Sci. 2024 Aug 30;15:1443691. doi: 10.3389/fpls.2024.1443691 (PMC11392766; doi:10.3389/fpls.2024.1443691)
Supplement: Supplementary file 1 [file DataSheet1.pdf]

Supplementary:

Supplementary Figure S1. Plant overexpression vector construction and identification of transgenic *Arabidopsis thaliana*. (A) Schematic diagram of the *SaPEPC1* gene being transferred to generate the overexpressing line. (B) Schematic diagram of *ZmPEPC1* gene being transferred to generate the overexpressing line. (C) Schematic diagram of *AtPEPC1* gene being transferred to generate the overexpressing line. (D) DL5000 DNA marker ladder. (E) pCAMBIA2300-*CaMV35S*::*SaPEPC1* enzyme cleavage map. (F) pCAMBIA2300-*CaMV35S*::*ZmPEPC1* enzyme cleavage map. (G) pCAMBIA2300-*CaMV35S*::*AtPEPC1* enzyme cleavage map. In E-F, lane 1 is the plasmid, and lane 2 is the result of enzyme digestion. (H) PCR analysis of *ZmPEPC1* overexpression in T3 generation. (I) PCR analysis of *SaPEPC1* overexpression in T3 generation. (J) PCR analysis of *AtPEPC1* overexpression in T3 generation. NT: non-transgenic plant. C−: negative control. C+: positive control. M: DNA marker.

Supplementary Figure S2. Chlorophyll fluorescence parameters of transgenic and wild type plants were analyzed under different light intensities. (A-C) Fv/Fm under normal light, intense light, and weak light. (D-F) Y(II) under normal light, intense light, and weak light. (G-I) Y(NO) under normal light, intense light, and weak light. (J-L) qN under normal light, intense light, and weak light. (M-O) qP under normal light, intense light, and weak light. WT, wide type; *SaP1* OE6, *SaP1* OE8: Two lines of *Arabidopsis* overexpressing *SaPEPC1* in T3 generation; *ZmP1* OE13, *ZmP1* OE17: Two lines of *Arabidopsis* overexpressing *ZmPEPC1* in T3 generation, *AtP1* OE3, and *AtP1* OE9: Two lines of *Arabidopsis* overexpressing *AtPEPC1* in T3 generation. Different lowercase letters above columns indicate significant differences between transgenic lines and WT under the same treatment conditions (p < 0.05). Values are means ± SE of six replicates.

Table. S1. Primer sequences used in the present study.

	Gene	Primer sequence5'-3'	
		Forward	Reverse
PCR	*SaPEPC1*	ATGGCAACTGTTAAGTTGGAGAGACT	TTAACCGGTGTTCTGCATTC
	ZmPEPC1	ACTACGAGGGCAAAGGAGACACGA	CTAGCCAGTGTTCTGCATGCC
	AtPEPC1	CAGGAAACAGCTATGAC	ACTTAACCGGTGTTTTGTAGACCAG
qRT-PCR	*AtPEPC1*	GCTGGATGAAATGGCGGTTG	CACCGCTTGGTTTACGCTTC
	AtRbcL	GCCCGTTCCAGGAGAAGAAA	TAAGCAGGAGGGATTCGCAG
	AtPPDK	GATAGCCCGAGAGCGAACAA	CACCAGTCCCTGAAGTGTCC
	AtCA1	TCAAATACGGTGGCGTTGGA	AAGCCCTTTGATCCCACCAC
	AtME2	AGGCTTGCCAGAAGTATGGG	CGAGAATCCGCTCACCATCA
	AtMDH	GCTGTTGCTGAGAATTGCCC	AGCCCTCACAACATCCAAAGT
	AtADG1	TCGTTGAAGTTCTCGCTGCT	TGCTCCTCGAACAACCACAA
	AtSUS5	TGGAAGCAAAGAGAGGGCTG	CTCCGGTCTTGTGCGGTAAA
	AtSPS1	AGCCAGCAGGGAGTGATTTG	AGAACCCGCCTCACAAAACT
	AtTPI	GAGGTTGTGGTTAGCCCTCC	TTCACAAGCATCTCCGCACT
	AtFBP	GGACCACACTGATGAGCCAA	TCCAGTGCTCAACACAAGCA
	Actin	GGTAACATTGTGCTCAGTGGTGG	AACGACCTTAATCTTCATGCTGC

* The Forward primers of *AtPEPC1* transgenic plants were identified using M13 Reverse primers contained in pCAMBIA-2300 overexpressing plant vectors.

Table. S2. The description of fluorescence parameters

O	Fluoorescene when all PSII RCs are open (≈to the minimal reliable recorded fluorescence)
J	Fluorescence at the J-step (2ms) of OJIP
I	Fluorescence at the I-step (30ms) of OJIP
P	Maximal recored fluorescence, at the peak P of OJIP
Vj	Relative variable fluorescence at the J-step (t = 2ms)
Vi	Relative variable fluorescence at time 30 ms (I-step) after start of actinic light pulse
Sm	Normalized area; it is related to the number of electron carriers per electron transport chain
ABS/RC	Absorption flux (of antenna Chls) per RC
DIo/RC	Total energy dissipation not trapped by the PSII reaction center
TRo/RC	Trapped energy flux (leading to Q_A reduction),per RC
ETo/RC	Electron transport flux (further than Q_A), per RC
ABS/CSm	The energy absorbed per unit leaf cross-section when reaching the fluorescence measurement maximum (t= tFM)
DIo/CSm	The heat dissipation per unit leaf section energy is obtained when the maximum value of fluorescence measurement is reached (t= tFM)
TRo/CSm	The energy flux captured by the PSII active reaction center per leaf section when the maximum fluorescence measurement is reached (t= tFM)
ETo/CSm	The energy flux of electron transport per unit leaf section when the maximum value of fluorescence measurement is reached (t= tFM)
PI abs	Performance index for energy conservation from photons absorbed by PSII until the reduction of intersystem electron acceptors
DF abs	Driving force (potential) for enery conservation from photons absorbed by PSII until the reduction of intersystem electron acceptors
Phi(Po)	Maximum quantum yield for primary photochemistry
Phi(Eo)	Quantum yield for electron transport

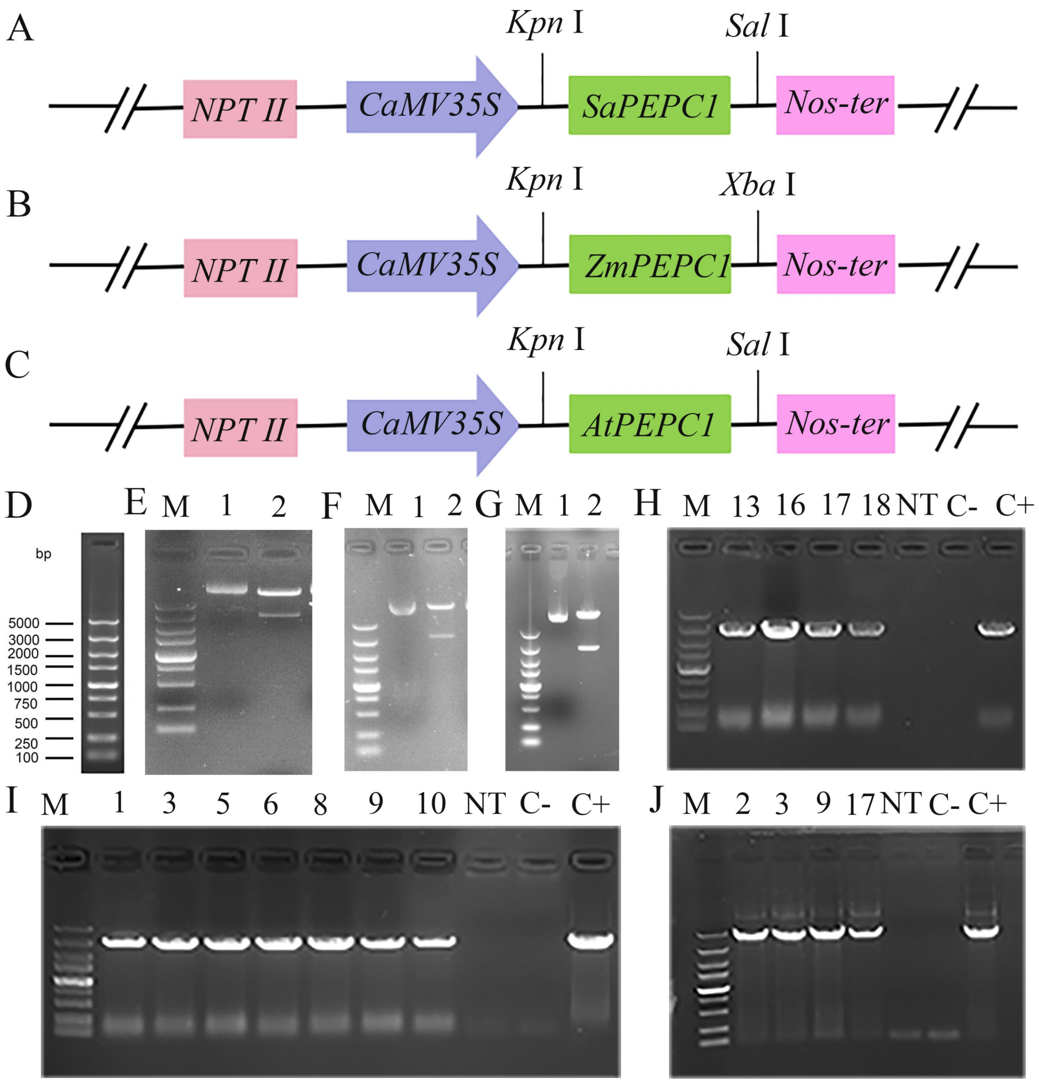
A
Kpn I
Sal I
NPT II
CaMV35S
SaPEPC1
Nos-ter
B
Kpn I
Xba I
NPT II
CaMV35S
ZmPEPC1
Nos-ter
C
Kpn I
Sal I
NPT II
CaMV35S
AtPEPC1
Nos-ter
D
bp
5000
3000
2000
1500
1000
750
500
250
100
E M 1 2
F M 1 2
G M 1 2
H M 13 16 17 18 NT C- C+
I M 1 3 5 6 8 9 10 NT C- C+
J M 2 3 9 17 NT C- C+

Fig. 1

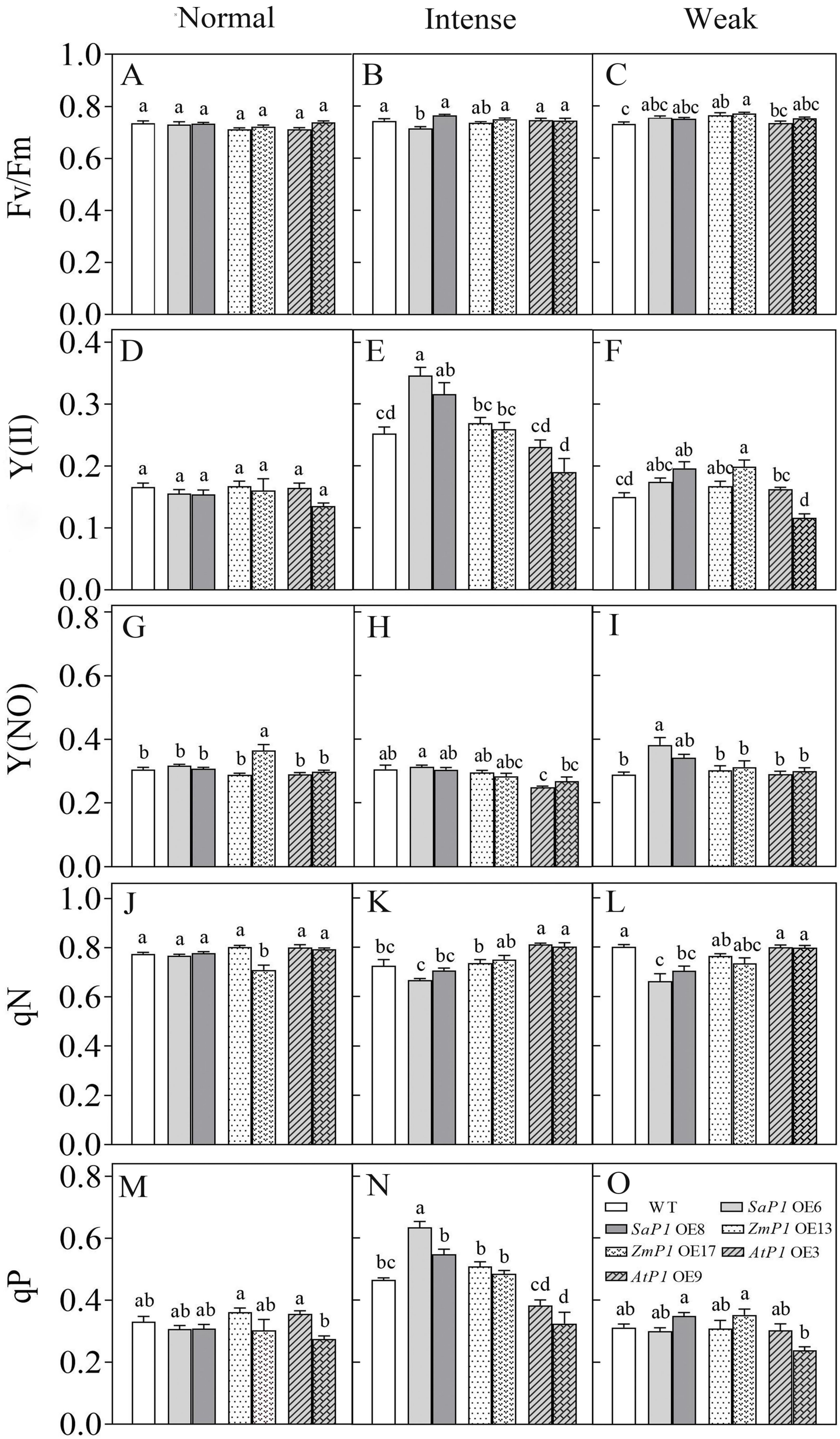
Normal
Intense
Weak
Fv/Fm
Y(II)
Y(NO)
qN
qP
WT
SaP1 OE6
SaP1 OE8
ZmP1 OE13
ZmP1 OE17
AtP1 OE3
AtP1 OE9

Fig. 2
